# The Oxytocin Neurons in the Paraventricular Nucleus Are Essential for Chronic Sleep Deprivation‐Mediated Anxiety‐Related Behaviors

**DOI:** 10.1111/cns.70465

**Published:** 2025-06-04

**Authors:** Yuxin Wang, Yifei Zhang, Yanchao Liu, Zhendong Xu, Jiyan Zhang, Like Wang, Yufeng Cang, Junbin Xin, Fuyu Han, Zhouhua Li, Chuanwei Hu, Xiangjie Kong, Yuchen Deng, Li Zhang, Hairong Wang, Haibo Xu, Ming Chen, Linlin Bi

**Affiliations:** ^1^ Department of Pathology Taikang Medical School (School of Basic Medical Sciences), Wuhan University Wuhan China; ^2^ Center for Pathology and Molecular Diagnostics Zhongnan Hospital of Wuhan University, Wuhan University Wuhan China; ^3^ Department of Radiology Zhongnan Hospital of Wuhan University, Wuhan University Wuhan China; ^4^ Department of Cardiology Zhongnan Hospital of Wuhan University, Wuhan University Wuhan China; ^5^ Institute of Myocardial Injury and Repair Wuhan University Wuhan China; ^6^ Hubei Provincial Clinical Research Center for Cardiovascular Intervention Wuhan China; ^7^ Guangdong Province Key Laboratory of Psychiatric Disorders Southern Medical University Guangzhou China

**Keywords:** anxiety, chronic sleep deprivation, oxytocin neurons, the paraventricular nucleus

## Abstract

**Aims:**

Sleep disorders increase the risk of anxiety disorders. The underlying mechanisms and potential targets remain poorly understood. Our research aimed to discover the essential role of oxytocin neurons in the paraventricular nucleus (PVN^OXT^ neurons) in regulating anxiety‐related behaviors following chronic sleep deprivation (cSD).

**Methods:**

In vivo optogenetic stimulation was used to regulate the activity of PVN^OXT^ neurons, and meanwhile, anxiety‐related behavioral tests were performed. Electrophysiological analysis was used to test neuronal synaptic transmission. In vivo fiber photometry was used to assess OXT release.

**Results:**

Through c‐Fos staining of the whole brain, we found that cSD decreased c‐Fos expression in the PVN and increased c‐Fos expression in the medial prefrontal cortex (mPFC). cSD promoted anxiety‐related behaviors mainly through inhibiting AMPAR‐mediated postsynaptic excitability of PVN^OXT^ neurons. Instant optogenetic activation of PVN^OXT^ neurons decreased anxiety‐like behaviors and promoted fear memory extinction by promoting oxytocin release into the mPFC. Similar to cSD, optogenetic long‐term low‐frequency (LTF) stimulation of PVN^OXT^ neurons promoted a prolonged inhibition of PVN^OXT^ neurons and increased anxiety‐like behaviors. Interestingly, short‐term high‐frequency stimulation (HFS) of PVN^OXT^ neurons displayed a long‐term potentiation of AMPAR‐mediated synaptic transmission of PVN^OXT^ neurons and could reverse cSD‐induced anxiety by promoting the OXT‐mediated inhibitory transmission of the mPFC.

**Conclusion:**

Our findings provide key mechanisms and promising deep brain stimulation strategies associated with synaptic plasticity for cSD‐induced obsessive anxiety.

## Graphical abstract

Chronic sleep deprivation (cSD) promoted anxiety‐related behaviors mainly through inhibiting AMPAR‐mediated postsynaptic excitability of PVN^OXT^ neurons. LTF simulation of PVN^OXT^ neurons could increase anxiety‐like behaviors by inhibiting the PVN^OXT^‐mPFC pathway. PVN^OXT^‐mediated inhibitory transmission of the mPFC was enhanced by HSF following cSD. HSF stimulation could reverse cSD‐induced anxiety by promoting the PVN^OXT^‐mPFC pathway.

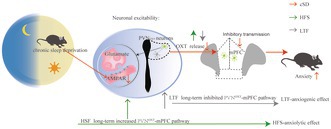

## 1 Introduction

Healthy sleep patterns are associated with reduced risks of anxiety disorders [1], whereas the presence of sleep disturbances has also been found to exacerbate the risk of developing anxiety disorders [2]. Control of insomnia significantly reduces anxiety disorders [3]. Despite these observations, the neural mechanisms through which sleep deprivation influences anxiety‐related emotional states remain poorly characterized. Elucidating these mechanisms is critical for understanding the pathophysiology of maladaptive emotions and developing targeted therapeutic strategies.

Oxytocin, a neuropeptide involved in mammalian birth and lactation, is produced mainly by the hypothalamus. Oxytocin is essential for human social cognition behavior, anxiety mood, and stress modulation. Many studies have been conducted on the connection between oxytocin levels in the central nervous system and psychiatric disorders such as schizophrenia, anxiety, depression, and autism spectrum disorders [4, 5]. In the magnocellular portion of the paraventricular nucleus (PVN), oxytocin‐containing magnocellular neurons project to the neurohypophysis, where they are discharged into the hypophyseal portal and systemic circulation. Previous investigations have identified a complex association between plasma oxytocin levels and anxiety induced by sleep deprivation [6]. Combined with the previous study, we hypothesize that sleep deprivation might affect anxiety emotion by modulation of the oxytocin pathway. However, the mechanism by which oxytocin regulates the emotional central nervous system or influences anxiety‐like behaviors has not been fully understood yet.

The PVN is a key node in anxiety regulation, with its neurosecretory parvocellular neurons contributing to stress responses and hypothalamic–pituitary–adrenal axis activation [2]. Previous studies found that oxytocin is a neuropeptide elaborated mainly by the PVN and the supraoptic nuclei (SON) [7, 8, 9]. Disorders of oxytocin secretion involve many psychiatric disorders including depression, anxiety, and schizophrenia, which have been recognized in many studies of animals and humans [7, 9]. Recent evidence further suggested that hypothalamic oxytocin neurons encode fear memory engrams [10]. Nevertheless, the involvement of PVN^OXT^ neurons in sleep deprivation‐mediated anxiety remains unclear.

There are some downstream brain regions, such as the medial prefrontal cortex (mPFC) [11] and the SON, receiving projections from PVN^OXT^ neurons [7, 12]. Oxytocinergic signaling in the PVN^OXT^‐mPFC circuit or PVN^OXT^‐SON plays a key role in pain sensory processing [7, 12]. Whether or how these neuronal circuits could modulate anxiety‐like behaviors after chronic sleep deprivation (cSD) was also unknown. Learning and memory‐related processes such as long‐term potentiation (LTP) and long‐term depression (LTD) of excitatory synaptic strength depend on the quick recruitment of extra α‐amino‐3‐hydroxy‐5‐methyl‐4‐isoxazolepropionate (AMPA) receptors (AMPARs) to the postsynaptic sites [13, 14]. Previous studies demonstrated that the firing of oxytocin neurons might be regulated mainly by AMPARs‐mediated neurotransmission [15]. Facilitation of AMPA‐evoked currents by the agonist appeared in oxytocin neurons. Glutamate controls the secretion of oxytocin through AMPARs [16]. Oxytocin could modulate spiking and synaptic plasticity [17]. Oxytocin in the anterior cingulate cortex attenuates neuropathic pain and emotional anxiety by inhibiting presynaptic LTP [18]. Sleep deprivation could decrease AMPAR binding in brain regions such as the hippocampus associated with learning and memory [19], but whether it affects synaptic plasticity in oxytocin neurons through AMPAR‐mediated pathways remained uninvestigated.

In this study, we applied cell‐type‐specific interventions to evaluate the role of PVN^OXT^ neurons on anxiety emotion following cSD. We then investigated the underlying mechanisms for modulating PVN^OXT^ neurons to cSD. Our research aimed to discover the complex roles of oxytocin neurons in regulating anxiety following cSD and provided mechanistic insights into maladaptive emotional states induced by cSD. We explored the optogenetic protocol for modulating synaptic plasticity, which might inspire the development of novel treatments for anxiety disorders involving deep brain stimulation to induce plasticity at relevant brain areas.

## 2 Methods

### 2.1 Animals

All animal experiments were conducted in strict adherence to guidelines by the National Research Council on the Care and Use of Laboratory Animals. C57BL/6J wild‐type mice (male, weighing 25–30 g, and aged 8–12 weeks) were randomly chosen and utilized for the experiments. All mice were housed under a controlled environment at a constant temperature of 22°C ± 2°C, 50%–60% humidity, and a 12 h/12 h light/dark cycle that began at zeitgeber time 0 (ZT 0). The animals were allowed unrestricted access to food and water. All behavioral tests were performed during the light cycle between 9:00 and 17:00 h. Both the number of mice utilized and suffering were kept to a minimum. All experimental procedures were carried out and approved by the Institute of Animal Care Committee at Wuhan University.

### 2.2 Virus Injection and In Vivo Optogenetic Stimulation

Mice (8–12 weeks old) were first anesthetized via intraperitoneal injection (ip) of sodium pentobarbital (50 mg/kg) (RWD Life Technology Co. Ltd., China), and positioned on a stereotaxic device. Ophthalmic ointment was administered to the mouse's eyes to stop corneal dryness and eye harm. Next, the scalp was shaved and cleaned with iodine and 70% alcohol, and then a longitudinal incision was made along the midline to reveal the skull. With the aid of the microscope, tiny holes were bored above the PVN (AP: −0.6 mm, ML: ±0.2 mm, DV: −4.8 mm) for viral injections (RWD Life Technology Co. Ltd.). The virus was injected at a rate of 10 nL/min using a micropipette fitted with the Auto‐Nanoliter Injector (Harvard). After the injection, the micropipette was held for 10 min to ensure virus diffusion and then gently removed.

For optogenetic manipulation of PVN‐OXT neurons, AAV2/9‐OXT‐ChR2‐mCherry, or AAV2/9‐OXT‐eNpHR3.0‐mCherry (PT‐2218, PT‐2812; 2 × 10^12^ genomic copies per mL; all these viruses packaged by Brain VTA Co. Ltd., Wuhan) or control viruses (PT‐0718; AAV2/9‐OXT‐mCherry; 2 × 10^12^ genomic copies per mL; packaged by Brain VTA Co. Ltd., Wuhan) were used. For the OXT neurotransmitter probe virus in the mPFC brain region, the virus (rAAV9‐hSyn‐OT1.8; 2 × 10^12^ genomic copies per mL in mixtures) or control viruses (AAV2/9‐DIO‐Ef1a‐EYFP; 3.9 × 10^12^ genomic copies per mL in mixtures) were injected, which all viruses were packaged by Brain Case Co. Ltd., Wuhan.

Next, PVN was injected with AAV2/9‐OXT‐mCherry, AAV2/9‐OXT‐ChR2‐mCherry, or AAV2/9‐OXT‐eNpHR3.0‐mCherry (50 nL for each microinjection, Brain VTA according to the Franklin and Paxinos Mouse Brain Atlas). Two weeks after the virus injection, the fiber ferrule (Inper Tech, China) was implanted 200 μm above the PVN (AP: −0.6 mm, ML: ±0.2 mm, DV: −4.6 mm), mPFC (AP: 1.95 mm, ML: ±0.25 mm, DV: −2.10 mm), or the SON (AP: −0.75 mm, ML: ±1.2 mm, DV: −5.1 mm). Behavioral tests were carried out 1 week after the fiber ferrule implantation, and then all animals were subjected to histological examination to confirm the site of viral expression. Any cases when viral expression occurred outside of the PVN area were omitted from the analysis. To activate the ChR2, a 473 nm laser's output power at the fiber's tip was set between 5 and 8 mW using an optical power meter (PM100D, Thorlabs, USA). Random pulses of 473‐nm light of 10‐ms width at 20 Hz were given to stimulate the PVN^OXT^ neurons optogenetically. For the activation of eNpHR3.0, a constant 589‐nm light was delivered into the PVN.

### 2.3 Oxytocin Receptor Antagonist

Mice received oxytocin antagonist L‐368899 1 h before all behavioral tests. Injections were administered intraperitoneally (ip) in all experiments (1 mg/kg/100 μL; Tocris Bioscience, UK) according to the previous studies [20].

### 2.4 Sleep Deprivation

Six hours (from 3 p.m. to 9 p.m.) of sleep deprivation was achieved from Day 1 to Day 7 using an automated sleep deprivation system, which consisted of an automatic cylindrical cylinder (PVC cylinder, height: 60 cm, width: 50 cm, weight: 5 kg), a small slow motor, and a metal bar (45 cm) connected to the small motor. To induce sleep deprivation, the bar was continuously rotated at a speed of approximately 3 rpm and randomly reversed in the direction of rotation to prevent subjects from gaining short sleep periods by adapting to the rotation pattern. A trained experimenter visually verified that the wooden bar was spinning during the sleep deprivation to ensure that mice had no chance to sleep.

### 2.5 Echocardiography

Echocardiography was conducted as previously described [21]. A small animal ultrasound imaging system, the VINNO D860 LAB (Beijing Yeeran technology Co. Ltd., China), was used to perform echocardiography. To measure the morphological and functional parameters, mice were anesthetized with 1.5% isoflurane. The probe measured the left ventricular short axis. M‐mode measurements provided information on heart rate (HR).

### 2.6 Elevated Plus Maze (EPM)

Mice were allowed to acclimatize to the testing room for 1 h and then put on the plus maze. The EPM was composed of three sections: two opposite open arms (50 × 10 cm each), two opposed closed arms (50 × 10 cm each) with 40 cm tall opaque walls, and a middle section (10 × 10 cm). Additionally, the maze was 50 cm above the ground. Mice were initially placed on the maze facing the closed arm, and then the behaviors were recorded for 5 min. In order to eliminate the smell left by the previous mouse, ethanol cotton was used to wipe the maze after each experiment. Computational software automatically measured the amount of time spent on the closed and open arms.

### 2.7 Open Field Test (OFT)

After an hour of acclimation to the testing environment, the mice were positioned in the box (50, 50, and 40 cm) and monitored for 5 min. Average movement speeds (mm/s), total lengths traveled (m), and time spent in the center of the testing box were measured. After the trial, the experiment box was cleaned to remove the scent of the preceding mouse. Less center‐area exploration is linked to anxiety‐related behaviors.

### 2.8 Fear Conditioning

As previously described [22], two types of fear conditioning shock chambers (Chamber A: 25 × 25 × 31 cm, plexiglass front and back with cross‐stripes pattern and aluminum side walls, grid‐floor connected to a scrambled shocker; Chamber B: 25 × 25 × 31 cm, square plastic chamber surrounded with white walls, plastic floor) and multiparameter activity monitors (JLBehv‐LG Instrument) were used [23]. The mice were handled twice daily for three consecutive days to habituate the manipulation before the fear training. One time‐continuous scrambling foot shock at 0.6 mA for 1 s served as the unconditioned stimulus (US), and a 70 dB sound served as the conditioned stimulus (CS). On the conditioning day, mice were transported individually from the housing chamber and placed into the fear conditioning chamber A. The animals were placed in an undisturbed state for 3 min. Then, they underwent four CS (70 dB; 30 s duration; 80 s intershock interval), with a US (0.6 mA; 1 s duration) to terminate each session. After spending two extra minutes in the training chamber, the mice returned to the colony room.

Mice were placed in chamber B, monitored for 3 min (pretone freezing), and then exposed to CS for 3 min (tone‐cued freezing) to test the tone‐cued fear memory expression. The freezing actions revealed the fear behaviors. These tone‐cued fear tests were conducted 6 days in a row for fear extinction. DigBehv, an animal behavior analysis software, calculated the percentage of freezing time in each period.

### 2.9 Electrophysiological Analysis

Mice were anesthetized with sodium pentobarbital (50 mg/kg), and their brains were taken out and immediately chilled using an artificial cerebrospinal fluid (ACSF) containing 220 mM sucrose, 1.3 mM CaCl_2_, 2.5 mM KCl, 1 mM NaH_2_PO_4_, 2.5 mM MgSO_4_, 10 mM glucose, and 26 mM NaHCO_3_. Brain tissues (300 μm) were sectioned using a VT‐1000S vibratome (Leica, Germany) and incubated at 34°C with normal ACSF (in mM: 126 NaCl, 1 MgSO_4_, 3 KCl, 1.25 NaH_2_PO_4_, 2 CaCl_2_, 10 glucose, and 26 NaHCO_3_) to recover for 30 min at 34°C followed by an additional 1 h at 25°C before recording. All solutions were saturated with 95% O_2_/5% CO_2_ (vol/vol). At 32°C–34°C, regular ACSF was superfused into the recording chamber at 2 mL/min. Neurons were recorded using pClamp 9.2 software (Axon Instruments) and whole‐cell voltage‐clamp methods (MultiClamp 700B amplifier, Digidata 1320A analog‐to‐digital converter). The following solution was filled into the glass pipettes: 105 mM K‐gluconate, 30 mM KCl, 10 mM HEPES, 10 mM phosphocreatine, 4 mM ATP‐Mg, 0.3 mM GTP‐Na, 0.3 mM EGTA, and 5 mM QX314 (pH 7.35, 285 mOsm). EPSCs were recorded in the presence of the GABA_A_R antagonist BMI (20 μM). To pharmacologically isolate AMPAR‐ or NMDAR‐mediated EPSCs, we blocked AMPAR with 20 μM CNQX or NMDAR with 100 μM AP5. One micromolar TTX was applied to the bath solution for recording mEPSCs. GABA_A_R‐mediated sIPSCs were recorded in the presence of CNQX (20 μM) and AP5 (50 μM). The resistance of pipettes was 3–5 MΩ.

When evoked synaptic currents were created, a two‐concentric bipolar stimulating electrode was placed about 100 mm from the recorded neurons in the PVN. The electrode's strength was calibrated to produce 50% of the maximum response. 0.2 ms pulses were delivered at 0.1 Hz and synchronized using a Mater‐8 stimulator (A.M.P.I). The holding potential for sIPSCs, mEPSC, or AMPAR‐eEPSCs was −70 mV. The holding potential was +40 mV for NMDAR‐eEPSCs. Data were collected when series resistance was controlled below 20 MΩ and fluctuations remained within 20% of the initial value.

### 2.10 Histology

After administering 50 mg/kg ip intraperitoneal sodium pentobarbital to sedate the mice, 30 mL of ice‐cold saline (0.9% sodium chloride) and 50 mL of ice‐cold 4% paraformaldehyde (PFA) in PBS were infused transcardially. After that, mouse brains were removed from the skull and postfixed for an additional night in 4% PFA. They were then dehydrated in PBS in 30% sucrose for 48 h at 4°C. Brain tissues were sectioned into slices that were 30 μm thick using a microtome (CM 1900, Leica). For c‐fos or OXT neuron staining, sections were soaked in a blocking solution (Beyotime), incubated for 20 min at room temperature (22°C ± 2°C), and rinsed three times in PBS for 10 min each. Next, either the rabbit polyclonal anti‐oxytocin‐neurophysin antibody (1:500, ab212193, ABcam, USA) or the rabbit polyclonal anti‐C‐fos antibody (1:10,000, ABE457, Millipore, USA) was used. The sections were incubated for 24 h with an anti‐rabbit c‐fos antibody or an anti‐rabbit oxytocin‐neurophysin antibody in a blocking solution at 4°C. After three more PBS washes (10 s each), the sections were incubated for 90 min at room temperature with the subsequent secondary antibodies: Thermo Scientific Alexa Flour 488 donkey anti‐rabbit IgG (1:500). Without c‐fos staining, sections were washed three times with PBS, directly stained with DAPI, mounted onto glass microscope slides, dried, and coated with mounting material. The images of sections were taken under an Olympus BX53 fluorescent microscope and processed with ImageJ 1.52V software.

### 2.11 Fiber Photometry

For OXT probe virus in mPFC brain region, the virus (rAAV9‐hSyn‐OT1.8; 2 × 10^12^ genomic copies per mL in mixtures, Brain Case Co. Ltd., Wuhan) was injected into the mPFC. The fiber optic cannulas were placed 0.1 cm above the mPFC (AP = 1.95 mm, ML = ±0.25 mm, DV = −2.20 mm).

The Neurophotometrics FP3002 fiber photometry system, which is available for purchase, was employed. In brief, the recording was accomplished by providing a 405 and 470 nm excitation light through the patch cord for calcium‐independent and calcium‐dependent fluorescence emission from rAAV9‐hSyn‐OT1.8. At the end of the patch cord, 75 mW of 405 and 470 nm light was produced by adjusting the excitation power. The bonsai open‐source software (Thinker Tech Nanjing Biotech Ltd.) was used to record at 30 Hz. The recorded traces were analyzed using our earlier research methodology [24].

### 2.12 Data Analysis

The number of experimental animals is indicated by “*n*.” Every dataset satisfied the normalcy assumption. An independent *t*‐test or a two‐way ANOVA followed by a repeated measure ANOVA was used for paired comparisons. The normality of distribution for continuous variables was assessed using the Shapiro–Wilk test. For normally distributed data, Student's *t*‐test and two‐way ANOVA followed by Tukey's post hoc test were used to compare means of two and multiple groups, respectively. Additionally, the Mann–Whitney *U*‐test and Kruskal–Wallis were employed to compare two or more groups of non‐normally distributed data, respectively. Statistical analyses were conducted using SPSS software (SPSS Inc.) throughout the study. All data were expressed as mean ± SEM. Significant values were defined as *p* < 0.05.

## 3 Result

### 3.1 The Acute Sleep Deprivation Produced Anxiolytic Effects, Which Quickly Disappeared, While Chronic Sleep Deprivation Promoted Anxiety‐Related Behaviors

**FIGURE 1:**
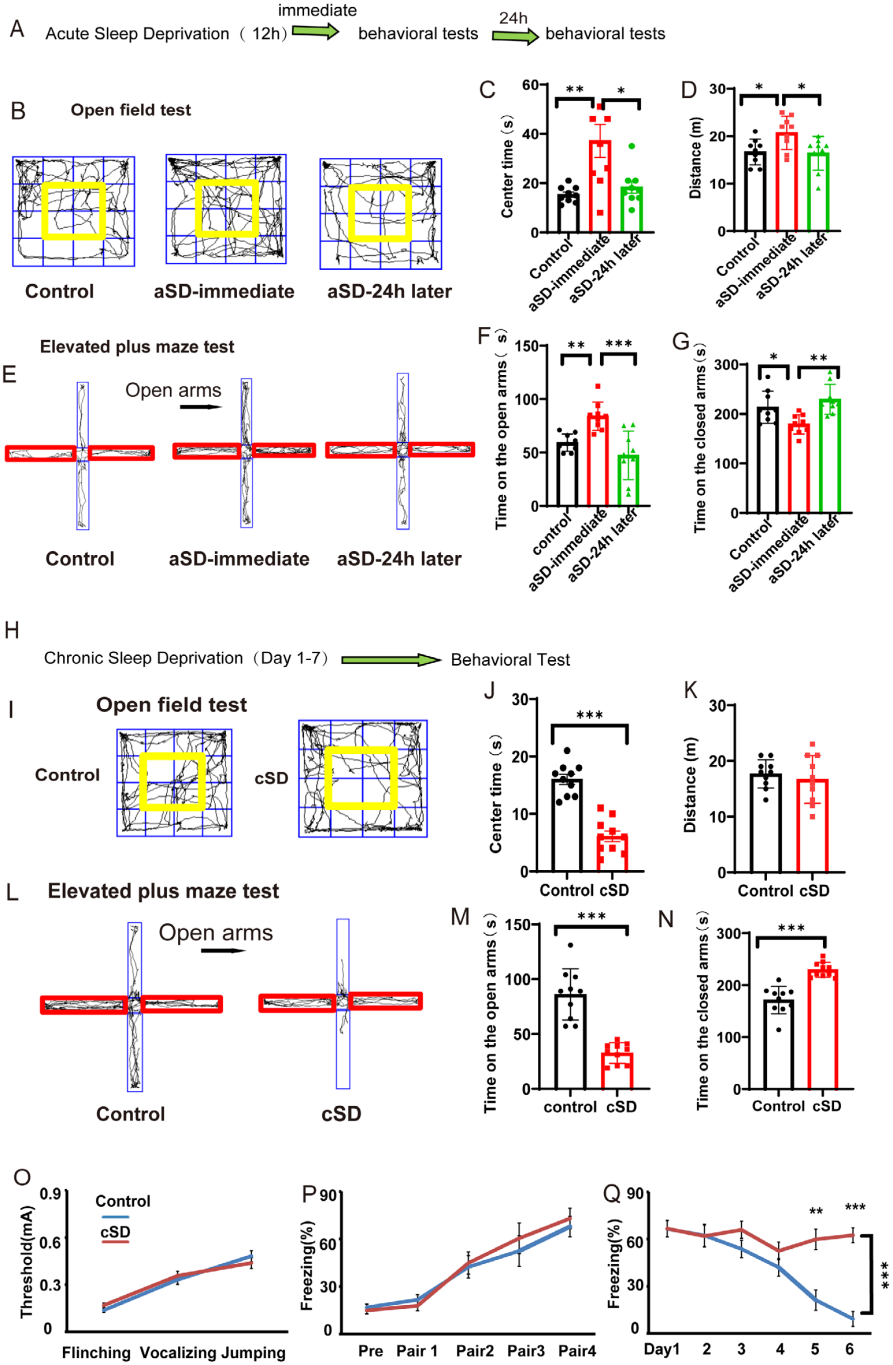
The acute sleep deprivation produced anxiolytic effects, which quickly disappeared, whereas chronic sleep deprivation promoted anxiety‐related behaviors. (A) A schematic diagram of the experiment. (B) Representative traces of open field test. The yellow frame represents the middle zone. (C) The statistical analysis results of the time spent in the center zone in the open field test. (D) Distance traveled in the open field test. (E) Representative traces of the elevated plus maze test. The red frame represents the closed arms. (F, G) Time spent on the open/closed arms in the elevated plus maze test. (H) A schematic diagram of the experiment. (I) Representative traces of open field test. The yellow frame represents the middle zone. (J) The statistical analysis results of the time spent in the center zone in the open field test. (K) Distance traveled in the open field test. (L) Representative traces of the elevated plus maze test. The red frame represents the closed arms. (M, N) Time spent on the open/closed arms in the elevated plus maze test. (O) Flinching, vocalization, and jumping thresholds of the experimental mice submitted to electric shock. (P) Freezing percentage during the fear training process (pre, no tone, and no shock; Pair1–4: 30 s tone + 1 s shock). (Q) Freezing percentage during the fear extinction process (3 min tone/day). For C–G, the statistical significance was determined using the one‐way RM ANOVA (*n* = 9/group). For I–N, the significance of the difference between groups was determined using an independent *t*‐test (for I–M, *n* = 10/group). The statistical significance for O–Q was determined using the two‐way RM ANOVA (*n* = 10/group). All error bars are SEM. *p < 0.05,**p < 0.01, ***p < 0.001.

Previous studies showed that acute sleep deprivation (aSD) had antidepressant effects [25, 26], but rare studies discussed the effect of aSD on anxiety‐like behaviors [27]. To test it, we subjected mice to 12 h aSD. Immediately after the aSD or 24 h after the aSD, mice underwent the open field test and the elevated plus maze test. We found that the aSD immediately increased time spent in the center zone of the open field box, which decreased to the normal level 24 h later (*F*_(2,24)_ = 7.792; *p* = 0.0032; Figure 1B,C). The aSD also increased the locomotion of mice, with normal recovery after 24 h (*F*_(2,24)_ = 4.899; *p* = 0.0388; Figure 1D). The aSD could also instantly increase open‐arm time and decrease the closed‐arm time during the elevated plus maze test, both of which disappeared 24 h after the aSD (*F*_(2,24)_ = 12.36 or *F*_(2,24)_ = 7.641; *p* = 0.0083 or *p* = 0.0392; Figure 1E–G). We found that aSD did not affect fear extinction (*p* > 0.05, Figure S8). Altogether, these results suggested that the aSD produced anxiolytic effects, but this effect quickly disappeared.

We investigated the potential impact of cSD on behaviors associated with anxiety. After 7 days of cSD, we tested the mice in the elevated plus maze, the open field, and the fear conditioning test. We discovered that the cSD decreased the time spent in the open field box's center zone (*t*(18) = 7.697, *p* < 0.0001; Figure 1I,J). The cSD did not affect the locomotion of mice either in the open field test (*t*(18) = 0.6336, *p* = 0.5343; Figure 1K) or in the elevated plus maze test (*p* > 0.05, Figure S5). The cSD could also decrease open‐arm time and increase closed‐arm time during the elevated plus maze test (*t*(18) = 6.74 or 6.111, both *p* < 0.0001; Figure 1L–N). We also found that the cSD did not affect the flinching, vocalization, and jumping thresholds of the experimental mice submitted to electric shock (*p* > 0.05, Figure 1O). The cSD group showed impaired fear extinction compared to the control group (*F*_(2,18)_ = 11.653; *p* < 0.001; Figure 1Q). Altogether, these results suggested that the cSD increased anxiety‐related behaviors and impeded the extinction of fear memories.

### 3.2 The cSD Affected the Excitability of PVN^OXT^ Neurons

**FIGURE 2:**
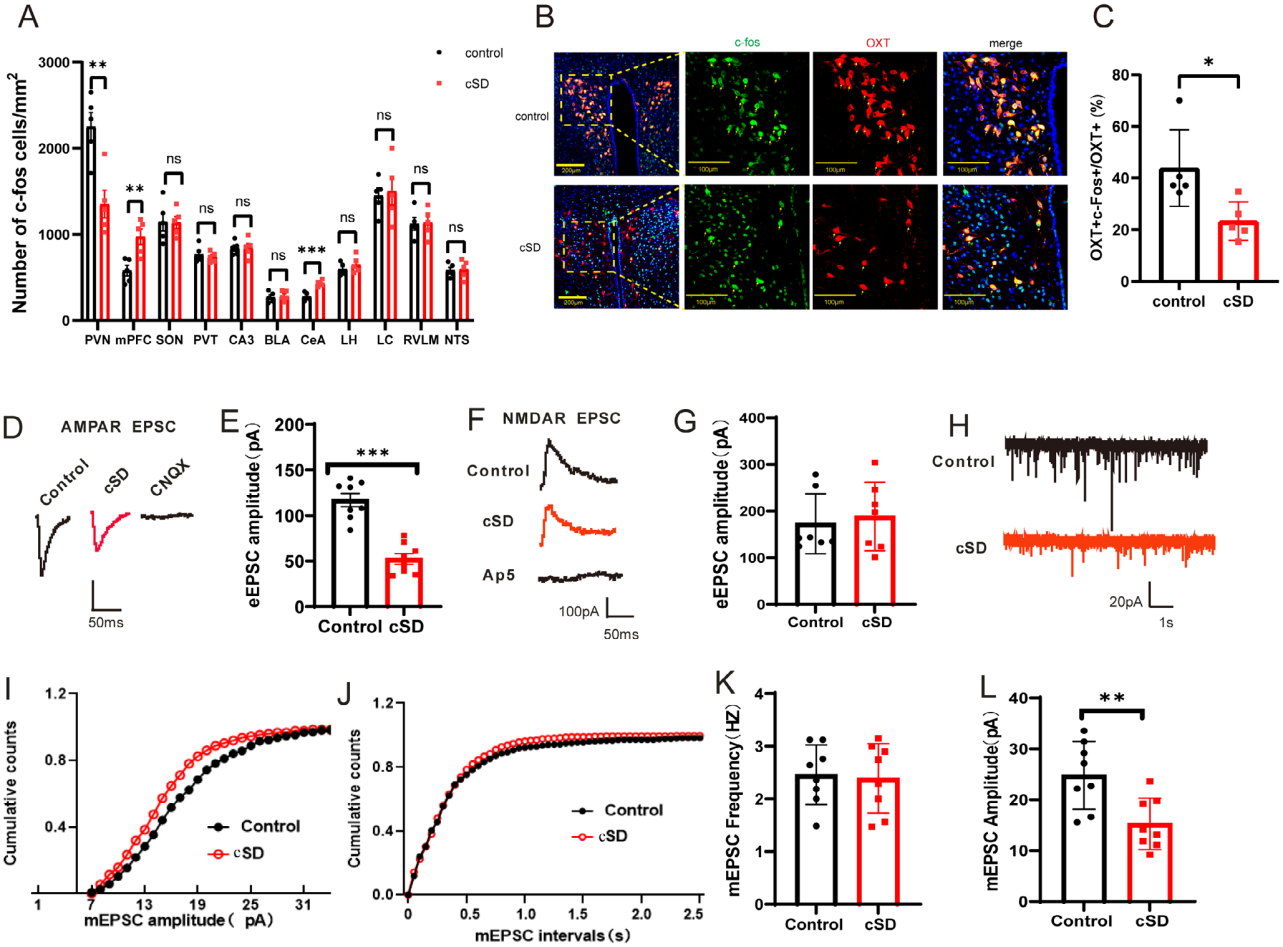
The effects of chronic sleep deprivation on the excitability of PVN^OXT^ neurons were analyzed. (A) Statistical analysis results of slice pictures showed the number of c‐Fos‐positive neurons in different brain areas in the control and cSD group (*n* = 5 mice/group). BLA: the basolateral amygdala; CeA: the central nucleus of the amygdala; LC: the locus coeruleus; LH: the lateral hypothalamus; NTS: the nucleus tractus solitaries; PVT: the paraventricular nucleus of the thalamus; RVLM: rostral ventrolateral medulla. (B) Co‐staining of PVN^OXT^ neurons with OXT antibody (red), c‐Fos antibody (green) and DAPI (blue) in the control group (up) and cSD group (down). Scale bar of picture on the left: 200 μm. Scale bar of three pictures on the right: 100 μm. (C) Statistical analysis results of slice pictures taken with confocal fluorescence microscopy showed the percentage of c‐Fos‐positive neurons in OXT‐positive neurons in the control and cSD group (*n* = 5 mice). AMPAR and NMDAR currents were evoked by holding membrane potentials at −70 mV and +40 mV, respectively. (D) Representative traces of AMPAR‐eEPSCs. (E) The peak amplitudes of the AMPAR‐eEPSC currents in the cSD mice were lower than those of the control mice. (F) Representative traces of NMDAR‐eEPSCs. (G) NMDAR currents did not differ between the two groups. (H) Representative traces of mEPSCs. (I) Cumulative plots of mEPSC amplitudes. (J) Cumulative plots of mEPSC frequencies. Summarized mEPSC frequencies (K) and amplitudes (L) (*n* = 8/group). Vertical bars represent the mean ± the SEM. Asterisks indicate significant differences from the relevant controls (*p < 0.05, **p < 0.01, ***p < 0.001, two‐tailed *t*‐test for A–C, D, F, and J–L).

To explore the brain regions affected by cSD, we stained whole brain sections following cSD. We found that cSD decreased c‐Fos expression in the PVN and increased c‐Fos expression in the mPFC and CeA. No significant changes in c‐Fos expression were found in other brain regions, such as PVT, LH, LC, RVLM, NTS, hippocampus, or BLA (Figure 2A and Figure S1). Disorders of oxytocin secretion involve many psychiatric disorders, including depression, anxiety, and schizophrenia, which have been recognized in many studies of animals and humans [7, 9]. The underlying mechanisms were not clear. Previous studies found that oxytocin is a neuropeptide elaborated mainly by the PVN and SON [7, 8, 9]. To verify the brain areas where oxytocin is expressed, co‐staining of PVN^OXT^ neurons with OXT antibody (red), c‐Fos antibody (green), and DAPI (blue) was performed after cSD. Consistent with the previous results, we found that OXT‐positive neurons were mainly distributed in the PVN and SON (Figure 2B and Figure S2A). The cSD decreased the percentage of c‐Fos‐positive neurons in OXT‐positive neurons in the PVN region (Figure 2B,C). However, the cSD did not affect the percentage of c‐Fos‐positive neurons in the SON region (Figure 2A). This result suggested that cSD reduced the activity of PVN^OXT^ neurons. We thus mainly explored the function of PVN^OXT^ neurons following cSD.

The cSD might affect anxiety emotion by modulation of the oxytocin pathway. We then measured excitatory postsynaptic currents (EPSCs) in PVN^OXT^ neurons in a whole‐cell configuration. One hundred millimolar AP5 was included in ACSF for the recording of AMPAR‐mediated evoked EPSC (AMPAR‐EPSC) and 20 mM CNQX was included in ACSF for the recording of NMDAR‐mediated evoked EPSC (NMDAR‐EPSC). AMPAR‐EPSCs were decreased in cSD mice compared to the control mice (*t*(14) = 6.857; *p* < 0.001; Figure 2D,E), but the NMDAR‐EPSCs did not differ between the two groups (*t*(14) = 0.4149; *p* = 0.686; Figure 2F,G). Moreover, CNQX and AP5 could block the AMPAR‐EPSC and NMDAR‐EPSC, respectively in Figure 2D,F. Lastly, we measured miniature excitatory postsynaptic currents (mEPSCs) from PVN^OXT^ neurons to investigate whether presynaptic or postsynaptic processes underlie lower AMPA‐EPSCs in the cSD mice. Figure 2H displays the representative traces of mEPSCs. Moreover, the findings of the mEPSC analysis are shown in Figure 2I–L. The amplitudes (*t*(14) = 3.241; *p* = 0.006; Figure 2I,L), but not the frequencies (*t*(14) = 0.227; *p* = 0.823; Figure 2J,K), of mEPSCs were decreased in the cSD group. To further test whether the GABAergic neurotransmission in the PVN^OXT^ neurons participated in cSD‐elicited responses, we measured spontaneous IPSCs (sIPSCs) in PVN^OXT^ neurons. Representative traces of the sIPSCs are shown in Figure S3A. The results of the sIPSC study are shown in Figure S3B,C. The cSD animals did not exhibit any differences in sIPSC frequencies or amplitudes compared to the control mice (*p* > 0.05). These results suggest that GABAergic neurotransmission of PVN^OXT^ neurons may not be affected by the cSD. Together, these findings suggest that the reduced excitability of PVN^OXT^ neurons may influence the cSD‐mediated anxiogenic process. The decreased excitability of PVN^OXT^ neurons might be due to the reduced AMPAR‐mediated postsynaptic mechanism.

### 3.3 Optogenetic Activation of PVN^OXT^ Neurons of Normal Mice Reduced Anxiety‐Like Behaviors and Promoted Fear Memory Extinction

**FIGURE 3:**
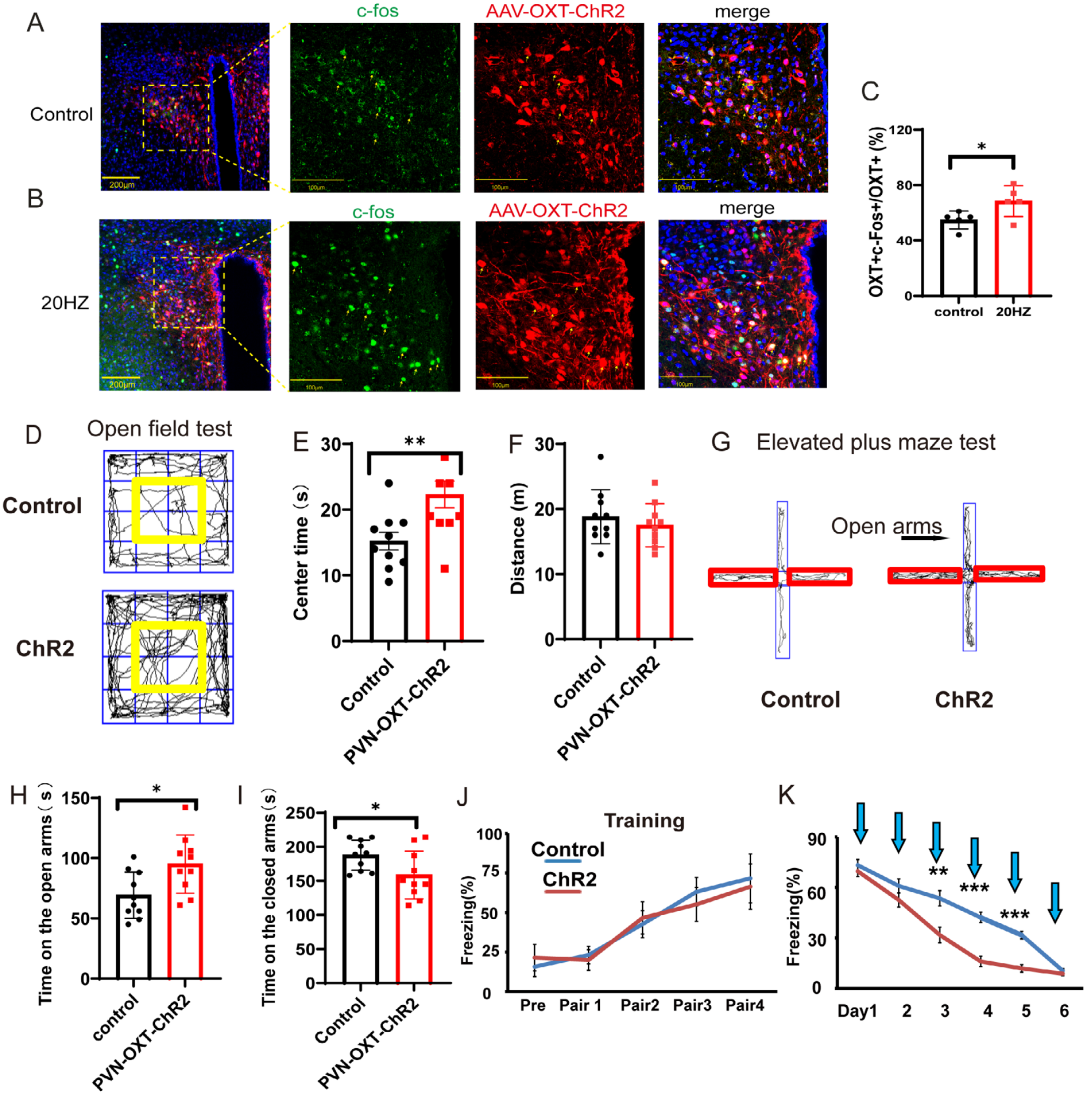
Optogenetic activation of PVN^OXT^ neurons of normal mice reduced anxiety‐like behaviors and promoted fear memory extinction. (A) Co‐staining of c‐Fos with AAV‐OXT‐ChR2‐mCherry‐expressing neurons without the optogenetic stimulation. Scale bar of the picture on the left: 200 μm. Scale bar of the three pictures on the right: 100 μm. (B) Co‐staining of c‐Fos with AAV‐OXT‐ChR2‐mCherry‐expressing neurons with 20 Hz blue light stimulation. Scale bar of picture on the left: 200 μm. Scale bar of three pictures on the right: 100 μm. (C) Statistical analysis results of slice pictures showed an increased percentage of OXT‐positive neurons in the 20 Hz blue light group. (D) Representative traces of open field test. The yellow frame represents the middle zone. (E) The statistical results of the time spent in the center zone in the open field test. (F) Distance traveled in the open field test. (G) Representative traces of the elevated plus maze test. The red frame represents the closed arms. (H, I) Time spent on the open/closed arms in the elevated plus maze test. (J) Freezing percentage during the fear training process (pre, no tone and no shock; Pair1–4: 30 s tone + 1 s shock). (K) Freezing percentage during the fear extinction process (3 min tone/day). Sections of PVN were prepared from adult mice which were transfected with AAV‐OXT‐ChR2‐mCherry. These sections were stained for c‐Fos (green) and DAPI (blue). The statistical significance was determined using the independent *t*‐test (*n* = 5/group). For D–I, the significance of the difference between groups was determined using the independent *t*‐test (*n* = 10/group). All error bars are SEM. *p < 0.05, **p < 0.01, ***p < 0.001.

Given that cSD suppressed PVN^OXT^ activity, we next tested whether optogenetic activation could rescue behavioral deficits. Representative immunohistochemical staining pictures showed that the AAV‐OXT‐hChR2‐mCherry virus was injected into the PVN (Figure 3B) and optogenetic stimulation of OXT neurons with a 473 nm laser (Figure 3B). Optogenetic activation of PVN^OXT^ neurons increased c‐Fos expression in AAV‐OXT‐ChR2‐mCherry‐expressing neurons, which proved the effectiveness of the virus (Figure 3C). To detect the specificity of the AAV‐OXT‐ChR2‐mCherry‐expressing neurons, we co‐stained OXT antibody with AAV‐OXT‐ChR2‐mCherry‐expressing neurons. We found that about 87.43% ± 5.29% of AAV‐OXT‐ChR2‐mCherry‐expressing neurons were OXT‐positive, which suggested a relatively good specificity of the virus (Figure S4).

Next, we had an optogenetic experiment. The AAV‐OXT‐hChR2‐mCherry virus was injected into the PVN, and optogenetic stimulation of OXT neurons with a 473 nm laser was delivered onto the PVN during the behavioral tests. We found that the optogenetic activation of PVN^OXT^ neurons increased time spent in the center zone of the open field box (*t*(18) = 2.883, *p* = 0.0099; Figure 3D,E). Besides, activation of PVN^OXT^ neurons increased time spent on the open arms and decreased time on the closed arms in the elevated plus maze test (*t*(18) = 2.640 or 2.226, *p* = 0.0166 or 0.039; Figure 3G–I). However, activation of PVN^OXT^ neurons did not affect the locomotion of mice (*p* > 0.05, Figure 3F). The freezing percentages during the fear training process were not different between the control and the ChR2 group (*p* > 0.05, Figure 3J). Activation of PVN^OXT^ neurons decreased freezing percentage during the fear extinction, especially on Days 3–5 (Day 3, *p* = 0.005; Days 4 and 5, both *p* < 0.001; Figure 3K). Altogether, these results suggested that enhanced activity of PVN^OXT^ neurons decreased anxiety‐related behaviors in the open field test and the elevated plus maze test. Activation of PVN^OXT^ neurons also promoted fear memory extinction.

### 3.4 PVN^OXT^ Neurons Regulated the Anxiety‐Like Behaviors Through the Release of Oxytocin

**FIGURE 4:**
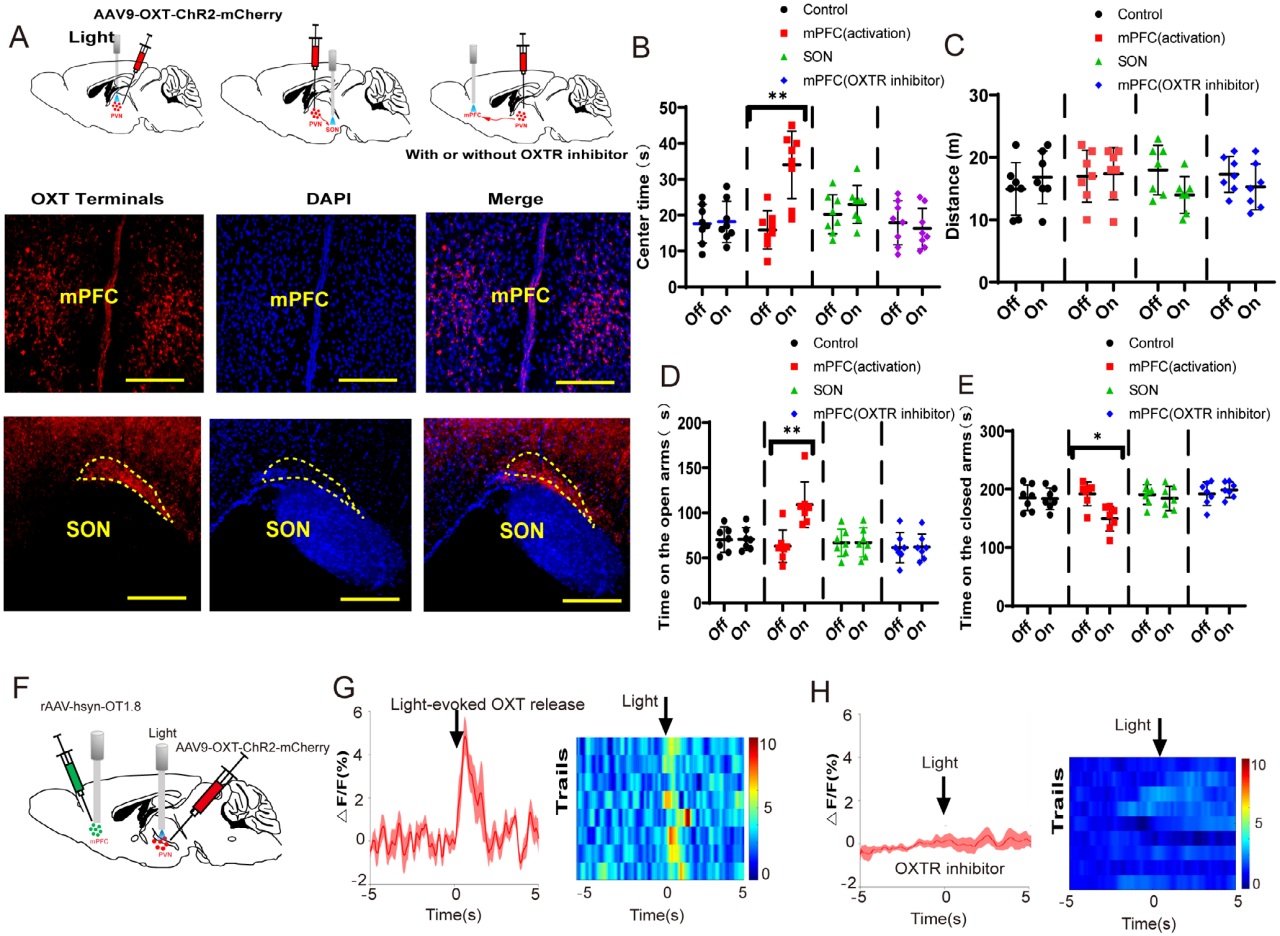
PVN^OXT^ neurons regulated the anxiety‐like behaviors through OXT receptors. (A) Upper panel, schematic illustration of the experimental protocols for B–E. For the control group, the AAV9‐OXT‐mCherry virus was injected into the PVN, and the 473 nm laser was on during the behavioral test. For the other three groups, the AAV‐OXT‐ChR2‐mCherry virus was injected into the PVN area. The 473 nm laser was delivered into the mPFC for the mPFC (activation) group and the mPFC (OXTR inhibitor, L‐368899) group. The same laser was delivered into the SON area for the SON group. Lower panel, representative immunohistochemical staining pictures showed virus‐expression terminals in the mPFC or in the SON, which originated from the PVN^OXT^ neurons (scale bar =200 μm). (B) The time spent in the center zone in the open field test (Off, light‐off; On, light‐on). (C) Locomotion in the open field test. (D, E) Time spent on the open/closed arms in the elevated plus maze test (*n* = 8/group). (F) The AAV‐OXT‐ChR2‐mCherry virus was injected into the PVN and the AAV carrying the GRAB sensor for OXT was injected into the mPFC. The optical fiber was implanted into the mPFC for the detection of OXT neurotransmitter signals. (G) The mean value (red trace) and color‐coded fluorescence intensity changes represented the average OXT release of all the transitions (light‐off to light‐on) without the OXT receptor inhibitor (OXTR inhibitor, L‐368899) (*n* = 8). (H) The mean value (red trace) and color‐coded fluorescence intensity changes represented OXT release from the light‐off phase to the light‐on phase with the OXTR inhibitor treatment (*n* = 9). The statistical significance was determined using the two‐way RM ANOVA. All error bars are SEM. *p < 0.05, **p < 0.01, ***p < 0.001.

To further investigate how activation of PVN^OXT^ neurons decreased anxiety‐related behaviors, we next explored the neural circuit and neurotransmission mechanisms of PVN^OXT^ neurons. We injected the AAV‐OXT‐ChR2‐mCherry virus into the PVN. The projecting terminals (red) were found in the SON, mPFC, PVT, LH, LC, RVLM, and NTS (Figure S2). However, cSD decreased c‐Fos expression in the PVN and increased c‐Fos expression in the mPFC (Figure 2A and Figure S1). No significant changes in c‐Fos expression were found in SON, mPFC, PVT, LH, LC, RVLM, or NTS (Figure 2A and Figure S1). We thus focus on the PVN^OXT^‐mPFC neuronal circuit. Though the SON and the mPFC have been found to receive dense projections from the PVN^OXT^ neurons and regulate pain sensory processing [7, 12], whether the PVN^OXT^‐mPFC or the PVN^OXT^‐SON regulates anxiety‐related behaviors was unknown. The mPFC and the SON received dense projections from the PVN^OXT^ neurons (Figure 4A). Optogenetic activation of PVN^OXT^ terminals in the mPFC could increase mice's time spent in the center during the open field test (*F*_(3,28)_ = 3.3; *p* = 0.0078; Figure 4B). Optogenetic activation of PVN^OXT^ terminals in the mPFC did not affect locomotion (*F*_(3,28)_ = 0.526; *p* = 0.668; Figure 4C). During the elevated plus maze test, optogenetic activation of PVN^OXT^ terminals in the mPFC could also decrease the time spent on the closed arms and increase the time spent on the open arms (*F*_(3,28)_ = 4.409 or 3.806; *p* = 0.0091 or 0.0156; Figure 4D,E). Nevertheless, in the elevated plus maze and open field tests, mice's anxiety‐like behaviors were unaffected by optogenetic stimulation of PVN^OXT^ terminals in the SON (Figure 4B–E).

To further test whether the OXT receptor (OXTR) antagonist could block the effect of optogenetic activation of PVN^OXT^ terminals in the mPFC, the oxytocin antagonist L‐368899 (which could pass the blood–brain barrier) was intraperitoneally injected into mice 1 h before all behavioral tests. Optogenetic activation of PVN^OXT^ terminals in the mPFC did not affect mice's anxiety‐like behaviors in the presence of L‐368899 (Figure 4B–E). We also assessed OXT levels in the mPFC by the fiber photometry, using targeted virus injections to express the GRAB sensor for OXT in the mPFC (Figure 4F–H). We discovered that the release of oxytocin into the mPFC could be enhanced by optogenetic activation of PVN^OXT^ neurons and that this release could be inhibited by the OXTR inhibitor L‐368899 (Figure 4G,H). Our findings suggested that by promoting the release of oxytocin into the mPFC, PVN^OXT^ neurons might have anxiolytic effects. This conclusion prompted us to investigate the neurophysiological mechanisms underlying this effect.

### 3.5 Long‐Term Low Frequencies (LTF) Stimulation of PVN^OXT^ Neurons Decreased the AMPAR‐Mediated Synaptic Currents of PVN^OXT^ Neurons and Increased Anxiety‐Like Behaviors

**FIGURE 5:**
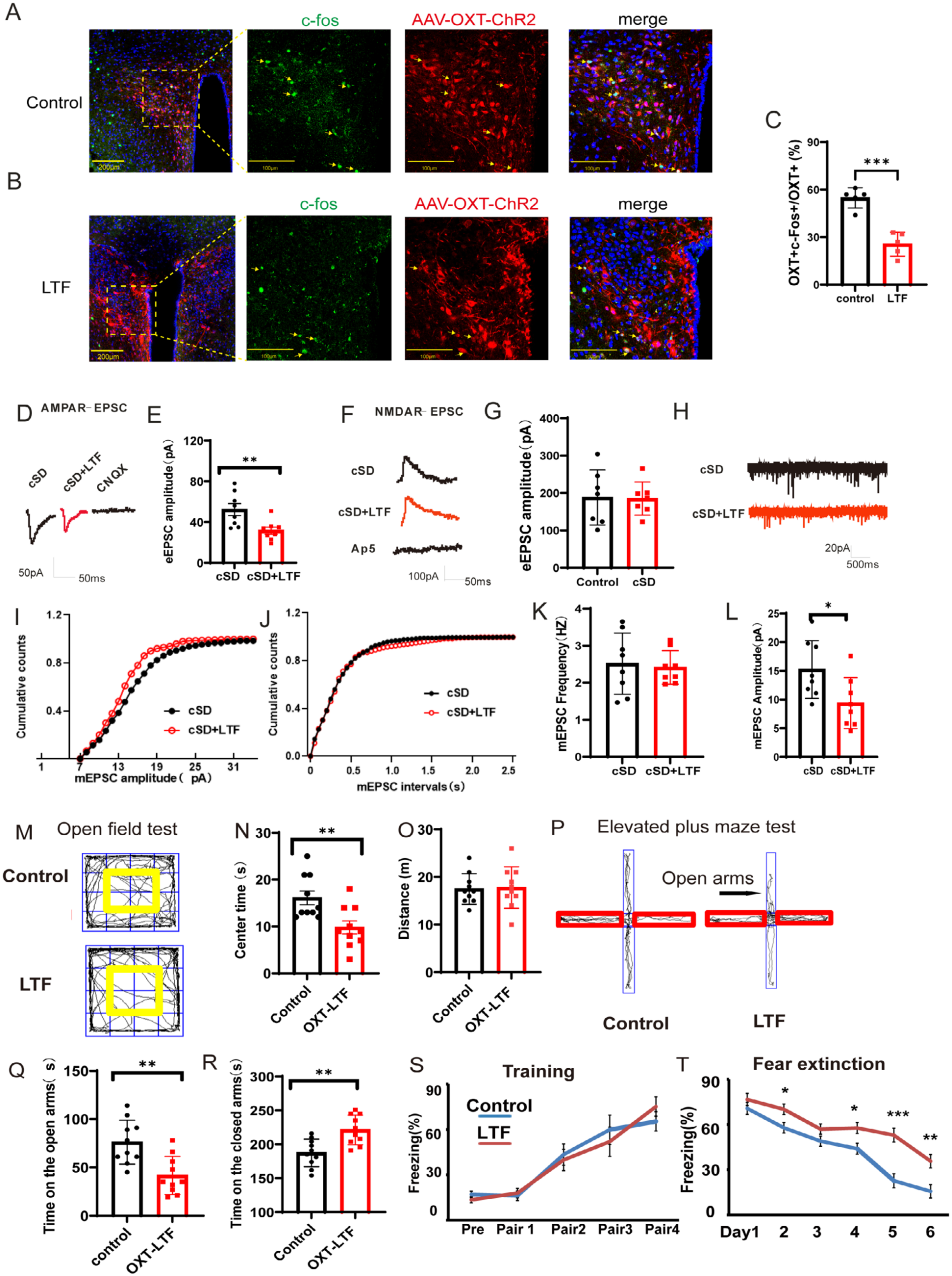
Long‐term low frequencies (LTF) stimulation of PVN^OXT^ neurons decreased the AMPAR‐mediated synaptic currents of PVN^OXT^ neurons and increased anxiety‐like behaviors. (A) Co‐staining of c‐Fos with AAV‐OXT‐ChR2‐mCherry‐expressing neurons without the optogenetic stimulation. Scale bar of the picture on the left: 200 μm. Scale bar of three pictures on the right: 100 μm. (B) Co‐staining of c‐Fos with AAV‐OXT‐ChR2‐mCherry‐expressing neurons with LTF stimulation. Scale bar of picture on the left: 200 μm. Scale bar of three pictures on the right: 100 μm. (C) Statistical analysis results of slice pictures showed a decreased percentage of OXT‐positive neurons in the LTF group. (D) Representative traces of AMPAR‐eEPSCs. (E) The peak amplitudes of the AMPAR‐eEPSC currents in the cSD + LTF mice were larger than those of the cSD‐D7 mice. (F) Representative traces of NMDAR‐eEPSCs. (G) NMDAR currents did not differ between the two groups. (H) Representative traces of mEPSCs. (I) Cumulative plots of mEPSC amplitudes. (J) Cumulative plots of mEPSC frequencies. Summarized mEPSC frequencies (K) and amplitudes (L) (*n* = 8/group). (M) Representative traces of open field test. The yellow frame represents the middle zone. (N) The statistical results of the time spent in the center zone in the open field test. (O) Distance traveled in the open field test. (P) Representative traces of the elevated plus maze test. The red frame represents the closed arms. (Q, R) Time spent on the open/closed arms in the elevated plus maze test. (S) Freezing percentage during the fear training process (pre, no tone and no shock; Pair1–4: 30 s tone + 1 s shock). (T) Tone‐elicited freezing percentage during fear extinction process (3 min tone/day). Sections of PVN were prepared from adult mice that were transfected with AAV‐OXT‐ChR2‐mCherry. These sections were stained for c‐Fos (green) and DAPI (blue). The statistical significance was determined using the independent *t*‐test (*n* = 5/group). For N–R, the significance of the difference between groups was determined using an independent *t*‐test (*n* = 10/group). The statistical significance for S and T was determined using the two‐way RM ANOVA (*n* = 8/group). All error bars are SEM. *p < 0.05, **p < 0.01, ***p < 0.001.

To test whether this oxytocin‐driven anxiolysis involves glutamatergic receptor regulation, we performed a whole‐cell configuration of PVN^OXT^ neuron. Then, we measured excitatory postsynaptic currents (EPSCs) to examine which type of glutamatergic receptors, AMPARs or NMDARs, contributes to the regulation of LTF. The AAV‐OXT‐hChR2‐mCherry virus was injected into the PVN. Three weeks after the virus injection, mice in the LTF group were photostimulated for 30 min with trains of 473 nm light (1 Hz, 4 ms, 900 pulses, 7 mW/mm^2^ at the PVN). One day after the LTF stimulation, brain slices were made, and thereafter, 100 mM AP5 was included in ACSF for the recording of AMPAR‐EPSC and 20 mM CNQX was included in ACSF for the recording of NMDAR‐EPSC. AMPAR‐EPSCs were decreased in cSD + LTF mice compared to the cSD mice (*t*(14) = 3.02; *p* = 0.009; Figure 5D,E), but the NMDAR‐EPSCs did not differ between the two groups (*t*(14) = 0.098; *p* = 0.923; Figure 5F,G). CNQX and AP5 could block the AMPAR‐EPSC and NMDAR‐EPSC, respectively, in Figure 5D,F. Lastly, by monitoring miniature excitatory postsynaptic currents (mEPSCs) from PVN^OXT^ neurons, we examined whether presynaptic or postsynaptic mechanisms underlie the decreased AMPA‐EPSCs in the LTF mice. Figure 5H displays the representative traces of mEPSCs. And Figure 5H–L are the analysis results of mEPSCs. The amplitudes (*t*(14) = 2.478; *p* = 0.027; Figure 5I,L), but not the frequencies (*t*(14) = 0.295; *p* = 0.772; Figure 5J,K), of mEPSCs were decreased in the cSD + LTF group. Altogether, these observations demonstrate that the LTF decreased the excitability of PVN^OXT^ neurons. The decreased excitability of PVN^OXT^ neurons might be due to the reduced AMPAR‐mediated postsynaptic currents.

We next test whether the long‐term low frequencies (LTF) stimulation of the PVN^OXT^ neurons could affect anxiety‐related behaviors and fear memory of normal mice. We found that the LTF stimulation of PVN^OXT^ neurons decreased time spent in the center zone of the open field box (*t*(18) = 3.105, *p* = 0.0061; Figure 5M,N). Besides, decreased time spent on the open arms and increased time on the closed arms were induced by the LTF stimulation in the elevated plus maze test (*t*(18) = 3.628 or 3.584, *p* = 0.0019 or 0.0021; Figure 5P–R). However, the LTF stimulation of PVN^OXT^ neurons did not affect the locomotion of mice either in the open field test (*p* > 0.05, Figure 5O) or in the elevated plus maze test (*p* > 0.05, Figure S5). We also found that the LTF stimulation did not affect the fear training process (*p* > 0.05, Figure 5S). The LTF stimulation of PVN^OXT^ neurons increased the freezing percentage during the fear extinction (*F*_(2,18)_ = 6.322, *p* = 0.008; Figure 5T). Altogether, these results suggested that the LTF stimulation of PVN^OXT^ neurons enhanced innate fear‐related behaviors of normal mice in the open field test and the elevated plus maze test. The LTF stimulation also impaired the fear memory extinction of normal mice.

### 3.6 Short‐Term High‐Frequency Stimulation (HFS) of PVN^OXT^ Neurons Could Decrease the Anxiety Level Induced by the cSD

**FIGURE 6:**
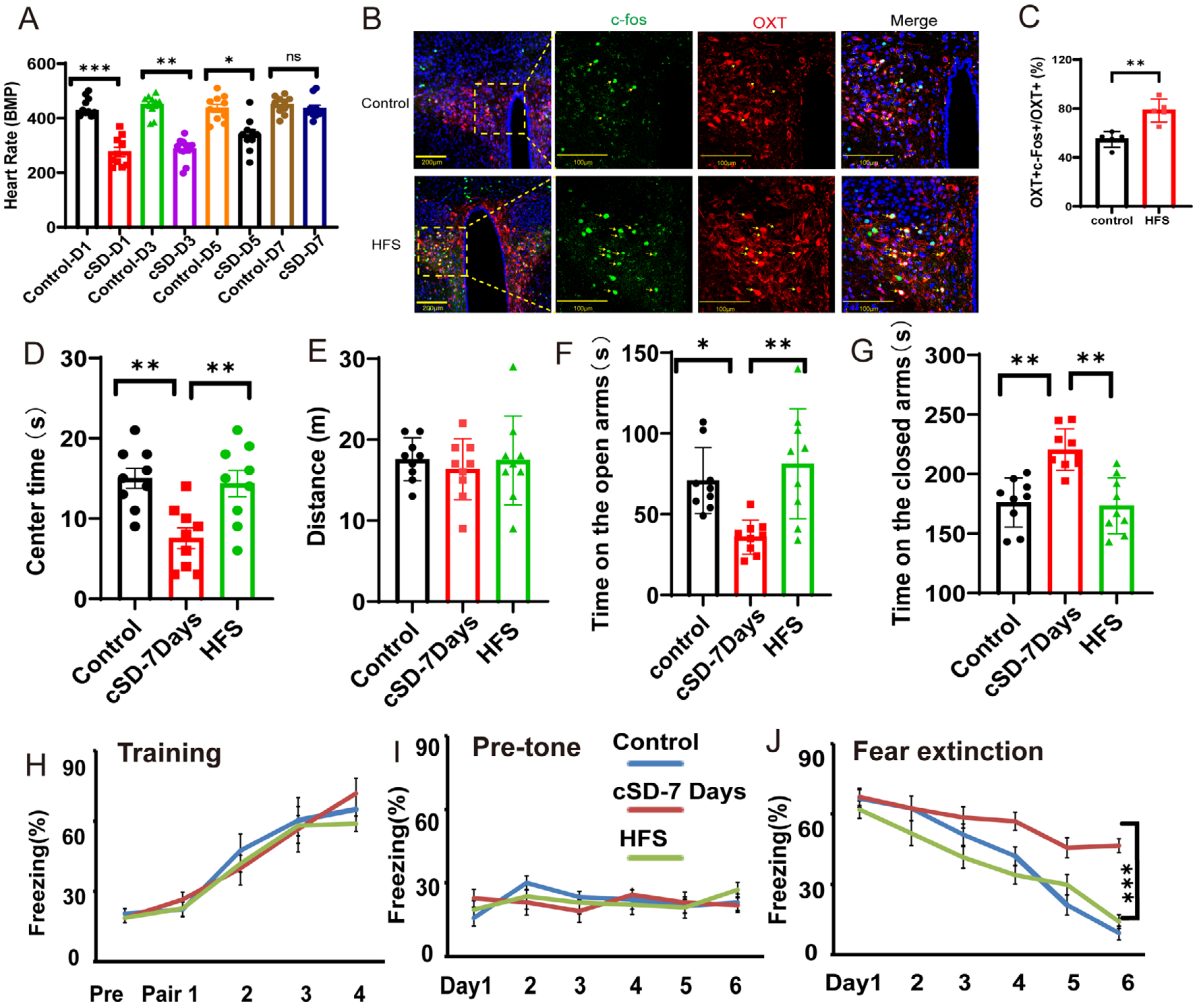
Short‐term high‐frequency stimulation (HFS) of PVN^OXT^ neurons could decrease the anxiety level induced by the cSD. (A) The heart rates were measured for seven consecutive days after the cSD. (B) Co‐staining of c‐Fos with AAV‐OXT‐ChR2‐mCherry‐expressing neurons without the optogenetic stimulation (up). Co‐staining of c‐Fos with AAV‐OXT‐ChR2‐mCherry‐expressing neurons with HFS stimulation (down). Scale bar of the picture on the left: 200 μm. Scale bar of the three pictures on the right: 100 μm. (C) Statistical analysis results of slice pictures showed an increased percentage of OXT‐positive neurons in the HFS group (*n* = 5 mice/group). (D) The statistical results of the time spent in the center zone in the open field test. (E) Distance traveled in the open field test. (F, G) Time spent on the open/closed arms in the elevated plus maze test. (H) The freezing percentage during the fear training process (pre, no tone and no shock; Pair1–4: 30 s tone + 1 s shock). (I) Freezing percentage was calculated before the tone during the fear extinction process (3 min pretone/day). (J) Tone‐elicited freezing percentage during fear extinction process (3 min tone/day). For A, H–J, the significance of the difference between groups was determined using a two‐way ANOVA test (*n* = 10/group). The statistical significance for D–G was determined using a one‐way ANOVA test (*n* = 9/group). All error bars are SEM. *p < 0.05, **p < 0.01, ***p < 0.001.

Sleep disorders have been recognized in recent years as health factors that can increase the risk for cardiovascular disease. Many neurons with neuroendocrine functions, such as oxytocin neurons, are essential for both the cardiovascular system and anxiety emotions. While intervening on oxytocin neurons in the PVN, attention should also be paid to the possible side effects on the peripheral cardiovascular system. Interestingly, we found that the heart rates recovered to normal levels 7 days after the cSD (Figure 6A). However, the increased anxiety emotion was still maintained at a high level more than 1 week after the cSD (Figure 6D–G).

High‐frequency stimulation of some functional brain regions could increase the activity of neurons, thereby rescuing abnormal conditions in mice or human [28, 29]. Therefore, we attempted to increase the activity of PVN^OXT^ neurons with high‐frequency blue light to improve the cSD‐induced anxiety‐related behaviors 7 days after the cSD. We injected AAV‐OXT‐hChR2‐mCherry virus into the bilateral PVN of wild‐type (WT) mice, and the HFS stimulation (~5 mW 473 nm light administered at five bouts of 100 Hz stimulation; 1 s/bout; 2 ms pulse width; with 15 s between bouts) was delivered into the PVN of cSD mice. We found that the HFS stimulation of PVN^OXT^ neurons 7 days after cSD increased the time spent in the center zone of the open field box, which reached the control level (*F*_(2,24)_ = 8.68, *p* = 0.0044; Figure 6D). Additionally, in the elevated plus maze test 7 days following the cSD, the HFS stimulation caused mice to spend more time on the open arms and less time on the closed arms when compared to the cSD group (*F*_(2,24)_ = 9.047 or 14.65, *p* = 0.0013 or < 0.001; Figure 6F,G). However, the HFS stimulation of PVN^OXT^ neurons did not affect the locomotion of mice either in the open field test (*p* > 0.05, Figure 6E) or in the elevated plus maze test (*p* > 0.05, Figure S5). Though the HFS stimulation did not change the fear training process and baseline freezing levels (both *p* > 0.05, Figure 6H,I), the HFS stimulation of PVN^OXT^ neurons decreased the freezing percentage during the fear extinction (*F*_(2,24)_ = 6.322, *p* < 0.001; Figure 6J). Altogether, these results suggested that the HFS stimulation of PVN^OXT^ neurons reversed the effect of the cSD on anxiety‐related behaviors. The HFS stimulation also promoted fear memory extinction after the cSD.

### 3.7 HFS of PVN^OXT^ Neurons Promoted the AMPAR‐Mediated Synaptic Currents of PVN^OXT^ Neurons

**FIGURE 7:**
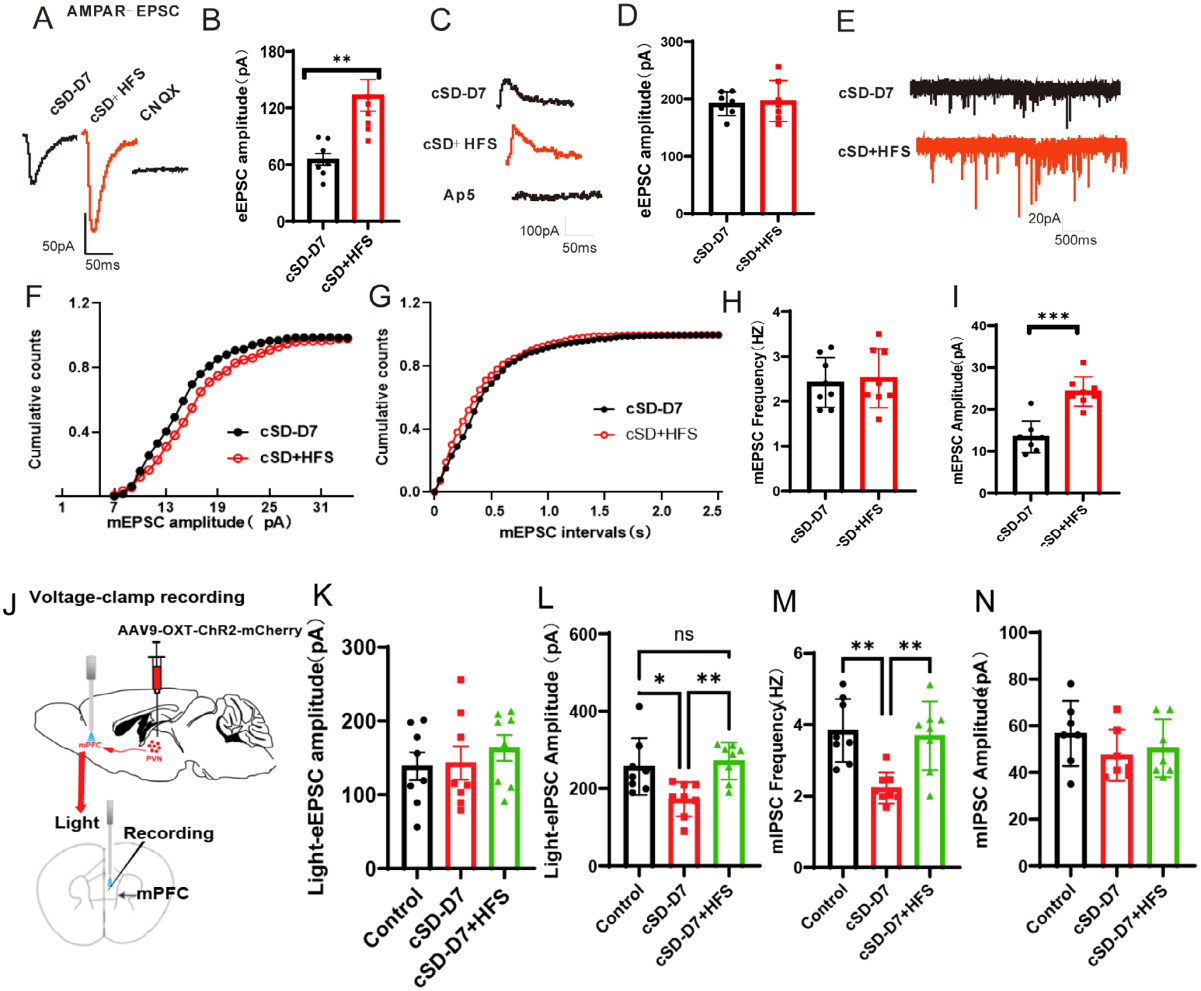
Short‐term high‐frequency stimulation (HFS) of PVN^OXT^ neurons promoted the AMPAR‐mediated synaptic currents of PVN^OXT^ neurons, and cSD did not affect the excitatory input to mPFC neurons from PVN neurons but led to decreased inhibition of mPFC neurons, which could be reversed by the HFS. (A) Representative traces of AMPAR‐eEPSCs. (B) The peak amplitudes of the AMPAR‐eEPSC currents in the cSD + HFS mice were larger than those of the cSD‐D7 mice. (C) Representative traces of NMDAR‐eEPSCs. (D) NMDAR currents did not differ between the two groups. (E) Representative traces of mEPSCs. (F) Cumulative plots of mEPSC amplitudes. (G) Cumulative plots of mEPSC frequencies. Summarized mEPSC frequencies (H) and amplitudes (I) (*n* = 8/group). (J) Experimental setup. The AAV‐OXT‐ChR2‐mCherry virus was injected into the PVN. HFS of PVN^OXT^ neurons was performed 3 weeks after the virus expression, and then the mPFC brain slices were made for the voltage‐clamp recording. The recording was performed in the mPFC when the PVN^OXT^ projecting terminals in the mPFC were optogenetically activated using a blue laser. cSD‐D7: 7 days after the cSD. cSD‐D7 + HFS: high frequency stimulating PVN^OXT^ neurons 7 days after the cSD. (K) Light‐eEPSCs were recorded in the presence of BMI (20 μM). Statistical analysis of light‐evoked EPSCs in mPFC neurons. (L) Light‐eIPSCs were recorded in the presence of CNQX (20 μM) and AP5 (100 μM). Statistical analysis of light‐evoked IPSCs in mPFC neurons. (M) Summarized data of mIPSC frequencies and mIPSC amplitudes (N). Holding potential: −70 mV. Data are expressed as the mean ± SEM. *n* = 8/group (8 cells recorded from 3 mice/group); *p < 0.05, **p < 0.01; ns, not significant; one‐way ANOVA test. Vertical bars represent the mean ± the SEM. The significance of the difference between groups was determined using an independent *t*‐test. Asterisks indicate significant differences from the relevant controls. *p < 0.05, **p < 0.01, ***p < 0.001.

To determine the molecular mechanisms underlying HFS‐mediated behavioral rescue, we examined which kind of glutamatergic receptors, NMDARs or AMPARs, contribute to the regulation of HFS. We recorded EPSCs in PVN^OXT^ neurons in a whole‐cell format. The AAV‐OXT‐hChR2‐mCherry virus was introduced into the PVN. Three weeks after the virus injection, the HFS was performed. One day after the HFS stimulation, brain slices were made, and thereafter, 100 mM AP5 was included in ACSF for the recording of AMPAR‐EPSC and 20 mM CNQX was included in ACSF for the recording of NMDAR‐EPSC. AMPAR‐EPSCs were significantly increased in cSD + HFS mice compared to the cSD‐D7 mice (*t*(14) = 3.802; *p* = 0.002; Figure 7A,B), but the NMDAR‐EPSCs did not differ between the two groups (*t*(14) = 0.302; *p* = 0.768; Figure 7C,D). CNQX and AP5 could block the AMPAR‐EPSC and NMDAR‐EPSC, respectively, in Figure 7A,C. Finally, we evaluated presynaptic or postsynaptic processes behind decreased AMPA‐EPSCs in the HFS mice by detecting miniature excitatory postsynaptic currents (mEPSCs) from PVN^OXT^ neurons. The typical traces of mEPSCs were given in Figure 7E. The mEPSC analysis results were shown in Figure 7F–I. The amplitudes (*t*(14) = 5.972; *p* < 0.001; Figure 7F,I), but not the frequencies (*t*(14) = 0.309; *p* = 0.762; Figure 7G,H), of mEPSCs were increased in the cSD + HFS group. We next checked whether HFS of PVN^OXT^ neurons could play an anxiolytic role by promoting the release of oxytocin into the mPFC. We assessed OXT levels in the mPFC by fiber photometry, using targeted virus injections to express the GRAB sensor for OXT in the mPFC (Figure S6A). Optogenetic activation of PVN^OXT^ neurons could induce the release of oxytocin into the mPFC in the control group (Figure S6B). HFS of PVN^OXT^ neurons significantly increased the release of oxytocin into the mPFC compared with the control group (Figure S6B,C). We also found that HFS of PVN^OXT^ neurons did not affect mice's anxiety‐like behaviors in the presence of OXTR inhibitor L‐368899 (Figure S6D–G). Altogether, our data suggested that the HFS improved the excitability of PVN^OXT^ neurons and increased the release of oxytocin into the mPFC region. The enhanced excitability of PVN^OXT^ neurons might be due to the increased AMPAR‐mediated postsynaptic neurotransmission.

To further explore the neurotransmission mechanism underlying high frequency stimulating PVN^OXT^ neurons that could regulate anxiety‐related behaviors following cSD, we injected the AAV‐OXT‐ChR2 virus into the PVN. HFS of PVN^OXT^ neurons was performed 3 weeks after the virus expression, and then the mPFC brain slices were made for the voltage‐clamp recording. The voltage‐clamp recording was performed in the mPFC when the PVN^OXT^ projecting terminals in the mPFC were optogenetically activated using a blue laser. We found that cSD did not affect the excitatory input to mPFC neurons from PVN neurons but led to significantly decreased inhibition of mPFC neurons, which could be reversed by the HFS (Figure 7J–N). The mIPSC frequencies, but not the amplitudes, were decreased by the cSD. Decreased mIPSC frequency suggests decreased presynaptic GABA release [30]. HFS of PVN^OXT^ neurons alleviated the cSD‐mediated decreased mIPSC frequencies. All the above results suggested that cSD reduced OXT secretion from the PVN^OXT^ terminals in the mPFC, thus inhibiting GABAergic transmission by decreasing the GABA release from the presynaptic terminals of PVN^OXT^ neurons. HFS of PVN^OXT^ neurons could promote OXT release. The released OXT promoted GABA release from the presynaptic terminals in the mPFC. Then the mPFC neurons that send anxiety signals were inhibited, and the anxiety‐like behavior was alleviated.

## 4 Discussion

For its role in controlling social and reproductive behavior, OXT—the “love hormone”—binds to specific OXT receptors (OXTRs) connected to the Gq/11 protein and the V1aR [31]. We studied the neural circuit mechanism of PVN^OXT^ neurons to modulate anxiety. We first verified that the effects of aSD and cSD on anxiety were the opposite: aSD produced anxiolytic effects, which quickly disappeared, but cSD promoted anxiety‐related behaviors. We next found that cSD promoted anxiety‐related behaviors mainly through inhibiting AMPAR‐mediated postsynaptic excitability of PVN^OXT^ neurons. Instant optogenetic activation of PVN^OXT^ neurons in normal mice decreased anxiety‐like behaviors and promoted fear memory extinction by promoting oxytocin release into the mPFC. In search of ways to modulate oxytocin neurons over the long term to treat the damage of cSD, we explored the different optogenetic stimulation modes. We found that optogenetic LTF stimulation of PVN^OXT^ neurons promoted a prolonged inhibition of AMPAR‐mediated synaptic transmission of PVN^OXT^ neurons and increased anxiety‐like behaviors. However, HFS of PVN^OXT^ neurons displayed a LTP of AMPAR‐mediated synaptic transmission of PVN^OXT^ neurons and could reverse cSD‐induced anxiety by promoting the OXT‐mediated inhibitory transmission of the mPFC. In adult rodents, LTP induction requires activation of Ca^2+^/calmodulin‐dependent protein kinase II (CaMKII). There is considerable evidence that an increase expresses it in the number of AMPA receptors (AMPARs) inserted into the postsynaptic membrane [32]. HFS‐induced enhancement of AMPAR function may involve the following mechanisms: (1) Ca^2+^‐dependent signaling cascades: HFS triggers the opening of L‐type voltage‐gated calcium channels (VGCCs), leading to Ca^2+^ influx, which activates CaMKII. This kinase subsequently phosphorylates the GluA1 subunit at the Ser831 site, promoting the insertion of AMPARs into the synaptic membrane [33, 34]. (2) Reorganization of the postsynaptic density: HFS may regulate the interaction between AMPARs and scaffold proteins through the PKC/PSD‐95 pathway, facilitating the establishment of LTP [35]. Therefore, cSD may suppress the VGCC‐Ca^2+^‐CaMKII‐AMPAR signaling pathway or weaken the PKC/PSD‐95‐mediated interaction between AMPARs and scaffold proteins, reducing synaptic transmission efficiency in PVN^OXT^ neurons. We will conduct further investigations to validate these hypotheses in future studies. Altogether, our studies suggested that intervening in PVN^OXT^ neurons could treat cSD‐mediated anxiety disorders.

PVN^OXT^ neurons manage anxiety‐like behavior by projecting to higher brain centers associated with anxiety, such as mPFC. We found that the mPFC and the SON received dense projections from the PVN^OXT^ neurons (Figure 4A). Optogenetic activation of PVN^OXT^ terminals in the mPFC, but not in the SON, could decrease mice's anxiety‐like behaviors. Activation of PVN^OXT^ promoted the release of oxytocin into the mPFC and optogenetic activation of PVN^OXT^ terminals in the mPFC did not affect mice's anxiety‐like behaviors in the presence of OXTR inhibitor L‐368899. We then found that the activity of PVN^OXT^ neurons was enhanced by the HFS stimulation (Figure 6B). HFS stimulation (long‐term excitation) of PVN^OXT^ neurons increased the release of oxytocin into the mPFC (Figure S6), inhibited GABAergic transmission by decreasing the GABA release from the presynaptic terminals of PVN^OXT^ neurons and significantly decreased the anxiety level induced by the cSD compared with the control group (Figure 6). We also found that HFS of PVN^OXT^ neurons did not affect mice's anxiety‐like behaviors in the presence of OXTR inhibitor L‐368899 (Figure S6). Our findings suggested that by promoting the release of oxytocin into the mPFC, PVN^OXT^ neurons could have anxiolytic effects following cSD.

To determine how cSD impairs PVN^OXT^ function to drive anxiety, we measured excitatory postsynaptic currents (EPSCs) and inhibitory postsynaptic currents (IPSCs) in PVN^OXT^ neurons in a whole‐cell configuration. AMPAR‐EPSCs, but the NMDAR‐EPSCs were decreased in cSD mice compared to the control mice. We also measured miniature excitatory postsynaptic currents (mEPSCs) and spontaneous IPSCs (sIPSCs) from PVN^OXT^ neurons to investigate whether presynaptic or postsynaptic processes underlie lower AMPA‐EPSCs in the cSD mice. The sIPSC frequencies or amplitudes of the cSD group were not changed compared to the control mice. The amplitudes but not the frequencies of mEPSCs were decreased in the cSD group. These findings suggest that the cSD reduced the excitability of PVN^OXT^ neurons and mediated the anxiogenic process. Because an mEPSC is the postsynaptic response to the release of an individual vesicle, a change in mEPSC amplitude indicates a change in receptor number [36]. The underlying physiological correlate for the increase in EPSC size is a postsynaptic upregulation of AMPARs at the membrane, which is accomplished through the interactions of AMPARs with many cellular proteins [36]. We thus inferred that the decreased excitability of PVN^OXT^ neurons might be due to the reduced postsynaptic AMPAR receptors.

The article just published [25] is about the effects of acute deprivation on depressive‐like behaviors, etc. The antidepressant effects of acute sleep loss were discussed in this paper, as well as in some other labs. However, they did not discuss the effect of acute sleep loss on memory, which might limit the significance of such research. We found that the effects of acute and chronic sleep disturbance were opposite. Though acute sleep disturbance (12 h, one time) could have an anxiolytic effect, it could also impair social memory (not in our paper this time, but we will discuss it in the future). We also found that the anxiolytic effect of aSD disappeared after 24 h. The benefits of acute sleep disturbance were inconsistent. So, we chose our research about chronic sleep disturbance.

Given the transient nature of aSD effects and the clinical relevance of cSD‐induced comorbidities (e.g., anxiety and cardiovascular dysfunction), we investigated the role of PVN^OXT^ neurons in mediating these outcomes. Sleep disorders have been recognized in recent years as health factors that can increase the risk for cardiovascular disease, heart attack, anxiety disorders, and so on. Many neurons with neuroendocrine functions, such as oxytocin neurons, are essential for both the cardiovascular system and anxiety emotions. When previous studies examined the functions of these neurons, they often only focused on their effects on the central nervous system or only on their effects on the periphery while ignoring all systemic effects. This paper suggested that while intervening on oxytocin neurons in the PVN, attention should also be paid to the possible side effects on the peripheral cardiovascular system. Interestingly, we found that cSD‐elicited cardiovascular responses, but not anxiety‐related behaviors, could recover 1 week later. Long‐term excitation of PVN^OXT^ neurons with HFS 1 week after cSD could reverse cSD‐induced anxiety by promoting the release of oxytocin into the mPFC, with minimal effect on the cardiovascular system (Figure S7). So, we recommended intervening in PVN^OXT^ neurons 1 week after the cSD to treat cSD‐mediated anxiety disorders.

We identified and validated a neuromodulatory circuit composed specifically of PVN^OXT^ neurons and explored the synaptic plasticity associated with cSD‐induced anxiety. Our findings will facilitate the investigation of pathophysiological mechanisms underlying fear‐related mental disorders, such as PTSD symptoms, and their potential treatment involving deep brain stimulation to induce plasticity at relevant brain areas.

## Author Contributions

Conceptualization: L.B., M.C., and H.X.; Methodology: Y.W., Y.Z., Y.L., Z.X., J.Z., L.W., Y.C., J.X., F.H., Z.L., C.H., X.K., Y.D., L.Z.; Investigation: Y.W., Y.Z., Y.L., Z.X., and X.K.; Writing – original draft: L.B. and Y.W.; Writing review and editing: M.C., L.B., H.X., and H.W.; Funding acquisition: L.B., M.C., and H.X.; Resources: M.C. and L.B.; Supervision: L.B., M.C., and H.X.

## Ethics Statement

The study complied with the Wuhan University Guide for the Care and Use of Laboratory Animals and the Animal Care Committee of Wuhan University (approval no. WAEF‐2023‐0293). Efforts were made to minimize animal suffering and to reduce the number of animals used.

## Conflicts of Interest

The authors declare no conflicts of interest.

## Supporting information


Figures S1–S8

## Data Availability

The data that support the findings of this study are available from the corresponding author upon reasonable request.
