# Supplementary material for: The Oxytocin Neurons in the Paraventricular Nucleus Are Essential for Chronic Sleep Deprivation‐Mediated Anxiety‐Related Behaviors
Source: CNS Neurosci Ther. 2025 Jun 4;31(6):e70465. doi: 10.1111/cns.70465 (PMC12137621; doi:10.1111/cns.70465)
Supplement: Supplementary file 1 — Figures S1–S8 [file CNS-31-e70465-s001.pdf]

Supplementary Method part

Intraperitoneal injection

OXTR inhibitor (L-368899, HY-108677, MecChem Express, USA; Atosiban, HY-17572, MecChem Express, USA) was used for intraperitoneal injection.

Supplementary Data

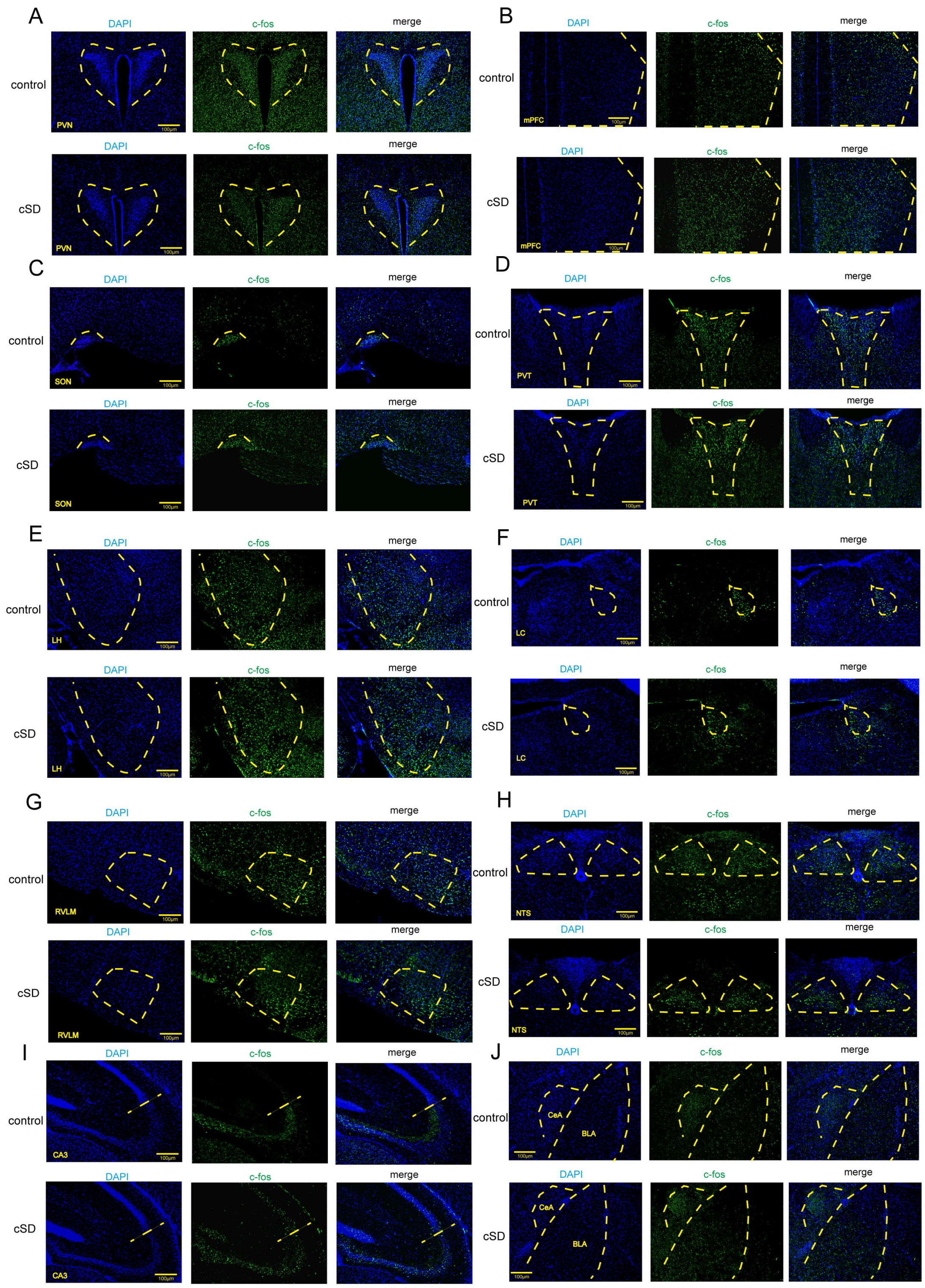

Figure S1. cSD decreased c-Fos expression in the PVN and increased c-Fos expression in the mPFC and CeA. No significant changes of c-Fos expression were found in other brain regions, such as PVT, LH, LC, RVLM, NTS, hippocampus or BLA.

Representative immunohistochemical co-staining pictures of c-Fos-positive neurons (green) and DAPI (blue) in the PVN(A), mPFC(B), SON (C), PVT(D), LH(E), LC(F), RVLM (G), NTS(H), hippocampus CA3(I), BLA and CeA(J). PVT: the paraventricular nucleus of the thalamus; LH: the lateral hypothalamus; LC: the locus coeruleus; RVLM, rostral ventrolateral medulla; NTS: the nucleus tractus solitaries; CeA: the central nucleus of the amygdala; BLA: the basolateral amygdala.

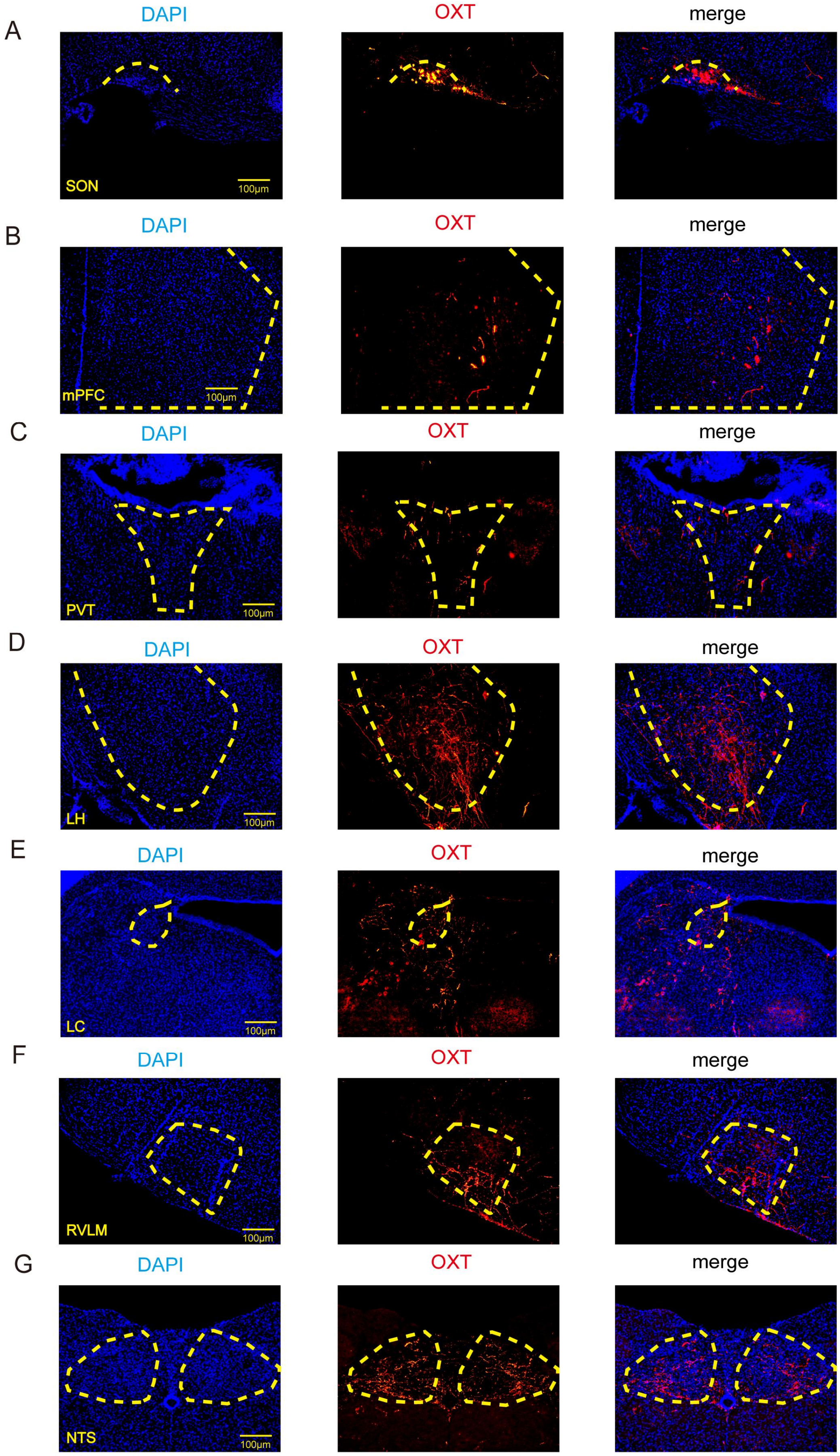
A
DAPI
OXT
merge
SON
100μm
B
mPFC
100μm
C
PVT
100μm
D
LH
100μm
E
LC
100μm
F
RVLM
100μm
G
NTS
100μm

Figure S2. OXT-positive neurons were mainly distributed in the PVN and SON(A). Terminals of PVN^{OXT} neurons were found in the Mpfc(B), PVT(C), LH(D), LC(E), RVLM(F) and NTS(G). (A)Representative immunohistochemical co-staining pictures of oxytocin antibody (red) and DAPI (blue) in the SON(A). (B-G) The AAV-OXT-ChR2-mCherry virus was injected into the PVN. The projecting terminals(red) were found in the mPFC(B), PVT(C), LH(D), LC(E), RVLM(F) and NTS(G).

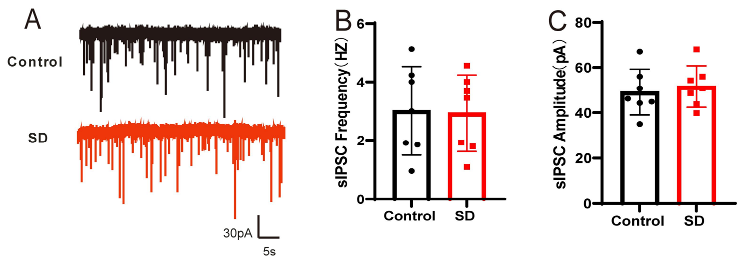

Figure S3. $GABA_A$ receptor-mediated synaptic currents of PVN^{OXT} neurons were not affected by chronic sleep deprivation.

(A) Representative mIPSCs traces are shown. Summarized mIPSC data showed no change of frequencies (B) or amplitudes (C) in the SD mice compared to the control mice (n=7/group). The statistical significance was determined using independent t-test. All error bars are s.e.m.

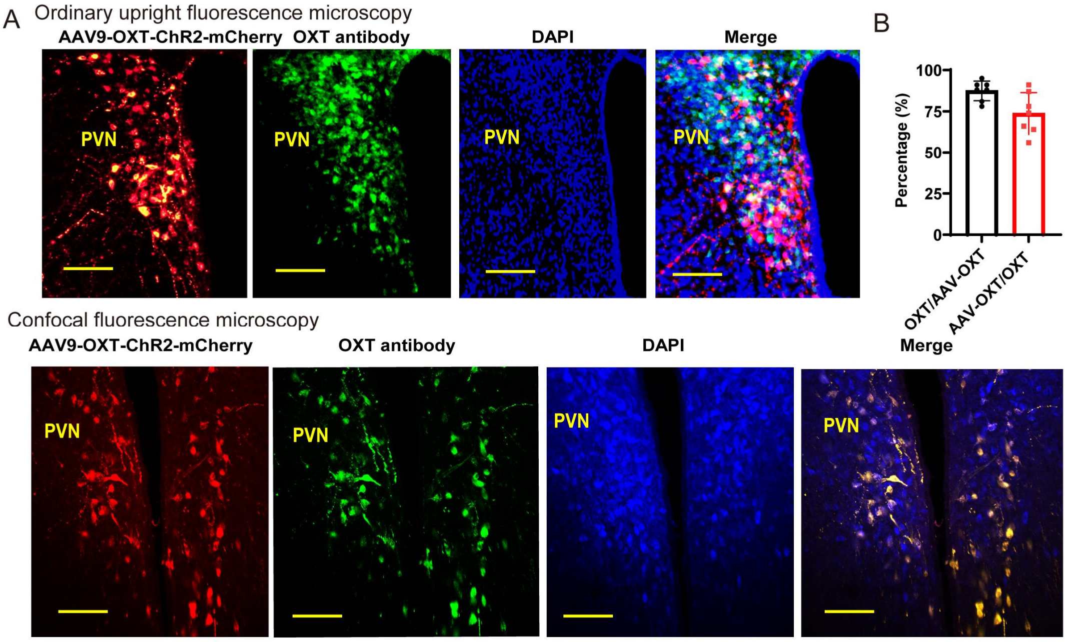

Figure S4. Sections of PVN were prepared from adult mice which were transfected with AAV-OXT-ChR2-mCherry.

(A) Co-staining of OXT antibody (green) with AAV-OXT-ChR2-mCherry-expressing neurons(red). Scale bars, 100μm. Upper panel, photos taken with ordinary upright fluorescence microscopy; lower panel, photos taken with confocal fluorescence microscopy. (B) Statistical analysis results of slice pictures taken with confocal fluorescence microscopy showed the percentage of OXT-positive neurons in AAV-OX-ChR2-mCherry-expressing neurons and AAV-OX-ChR2-mCherry-expressing neurons in OXT-positive neurons (n=7 mice).

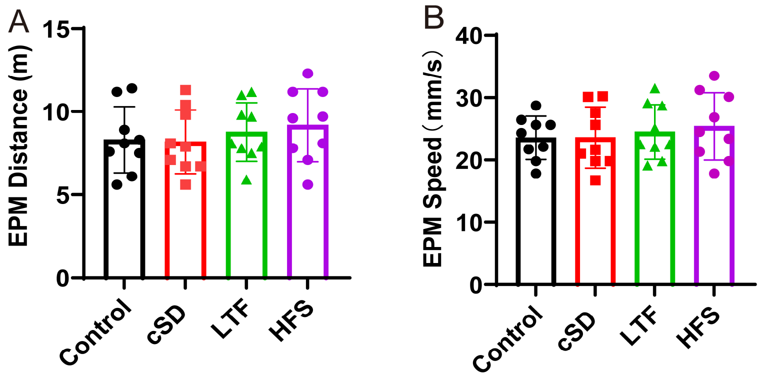

Figure S5. No changes in the locomotion were found between the control, the cSD group, the LTF group and the HFS group.

(A) Distance traveled in the elevated plus maze test. (B) The average speed in the elevated plus maze test. (n=9/group). The statistical significance was determined using a one-way ANOVA test. All error bars are s.e.m.

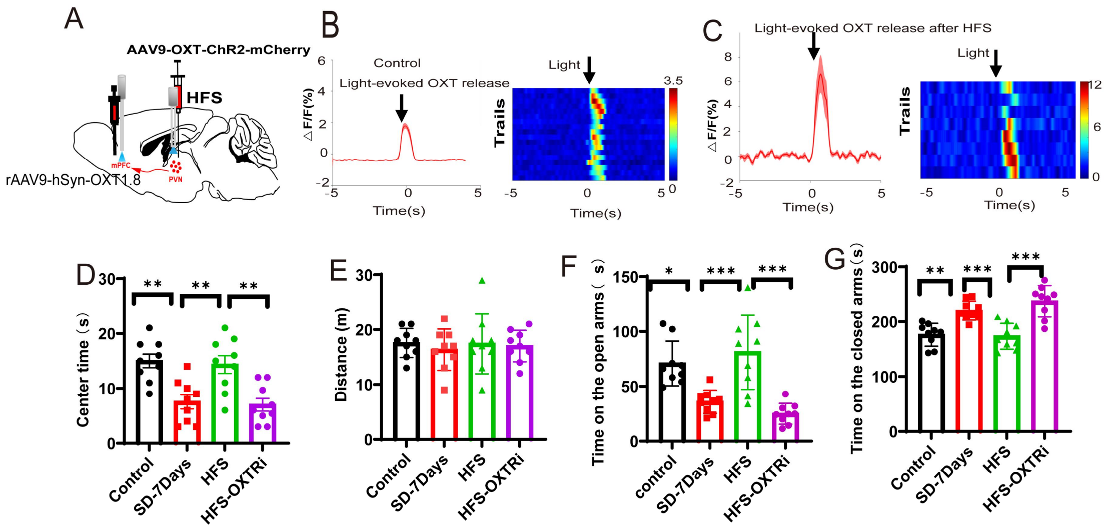

Figure S6. HFS of PVN^{OXT} neurons decreased anxiety induced by the cSD through increasing the release of OXT transmitter.

(A) Schematic illustration of the experiment protocols for B-4C. The AAV9-OXT-mCherry virus was injected into the PVN, and the HFS was delivered into the PVN. The AAV carrying the GRAB sensor for OXT was injected into the mPFC. The optical fiber was implanted into the mPFC for the detection of OXT neurotransmitter signals. 473nm light-evoked OXT release was measured in the mPFC before or after HFS. (B) The mean value (left panel) and color-coded fluorescence intensity changes (right panel) represented the average OXT release of all the transitions (light-off to light on) in the mPFC of cSD mice without HFS(n=18). (C) The mean value (left panel) and color-coded fluorescence intensity changes (right panel) represented OXT release from the light-off phase to the light-on phase in the mPFC of cSD mice after HFS(n=8). (D)The time spent in the center zone in the open field test (OXTRi，OXT receptor inhibitor). (E) Locomotion in the open field test. (F, G) Time spent on the open/closed arms in the elevated plus maze test(n=8/group). The statistical significance was determined using a one-way ANOVA test. All error bars are s.e.m. *$p < 0.05$, **$p < 0.01$, ***$p < 0.001$.

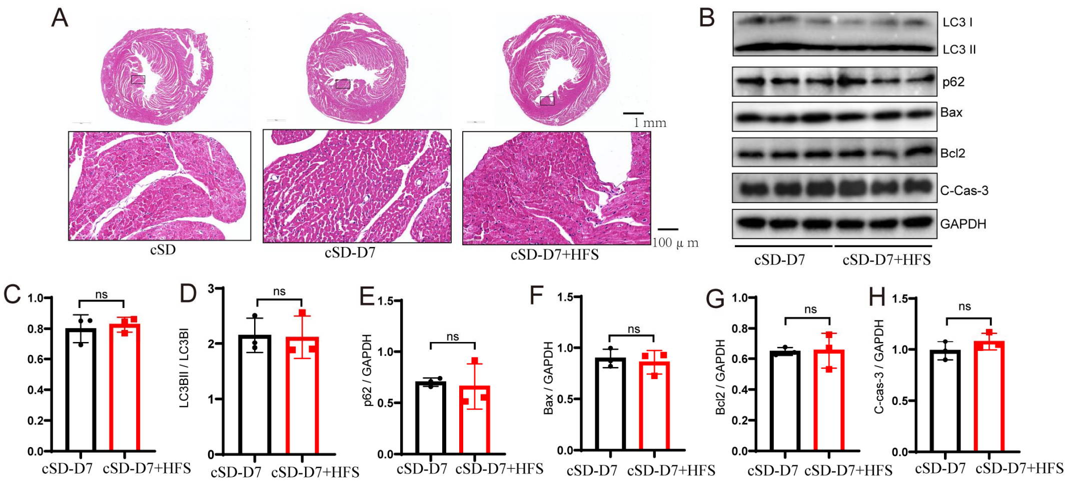

Figure S7. Short-term high frequency stimulation (HFS) of PVN^{OXT} neurons did not affect the morphological structure of myocardial cells and the autophagy and apoptosis of cardiac tissue.

(A) Hematoxylin & eosin (H&E) images of crosscut section heart of control, cSD–D7 and cSD-D7+HFS mice (scale bar = 1 mm or 100 μm). (B-H) Representative western blot image and quantification of LC3-I, LC3-II, p62, Bax, Bcl2, and c-Cas-3 SOD2 in control, cSD-D7 and cSD+HFS mouse hearts (n=3/group). Vertical bars represent the mean±the SEM. The significance of difference between groups was determined using independent t-test. Asterisks indicate significant differences from the relevant controls. *$p < 0.05$, **$p < 0.01$, ***$p < 0.001$.

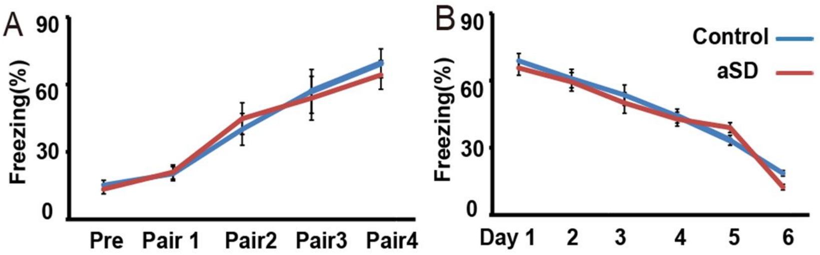

Fig S8. Acute sleep deprivation had no effect on fear conditioning.

(A) Freezing percentage during the fear training process (pre, no tone and no shock; Pair1-4: 30s tone+1s shock). (B) Freezing percentage during the fear extinction process (3min tone/day). The statistical significance was determined using the two-way RM ANOVA (n=10/group). All error bars are s.e.m.
